# A sphingolipid-derived paclitaxel nanovesicle enhances efficacy of combination therapies in triple-negative breast cancer and pancreatic cancer

**DOI:** 10.1038/s43018-025-01029-7

**Published:** 2025-08-21

**Authors:** Zhiren Wang, Wenpan Li, Yanhao Jiang, Teng Ma, Mengwen Li, Shuang Wu, Tuyen Ba Tran, Leyla Estrella Cordova, Ethan Lin, Aaron James Scott, Jennifer Erdrich, Joyce Schroeder, Pavani Chalasani, Jianqin Lu

**Affiliations:** 1 Skaggs Pharmaceutical Sciences Center, Department of Pharmacology & Toxicology, R. Ken Coit College of Pharmacy, The University of Arizona, Tucson, AZ USA; 2 Clinical and Translational Oncology Program, The University of Arizona Cancer Center, Tucson, AZ USA; 3 Division of Hematology and Oncology, Department of Medicine, College of Medicine, The University of Arizona, Tucson, AZ USA; 4 Department of Surgery, Division of Surgical Oncology, The University of Arizona College of Medicine, Tucson, AZ USA; 5 Department of Molecular and Cellular Biology, The University of Arizona, Tucson, AZ USA; 6 Division of Hematology and Oncology, George Washington Cancer Center, George Washington University, Washington, DC USA; 7 BIO5 Institute, The University of Arizona, Tucson, AZ USA; 8 Southwest Environmental Health Sciences Center, The University of Arizona, Tucson, AZ USA

**Keywords:** Cancer, Cancer therapy, Drug delivery, Nanotechnology in cancer

## Abstract

Taxol and Abraxane, the US Food and Drug Administration-approved paclitaxel (PTX) formulations, have revealed hypersensitivity due to excipients and mediocre efficacy due to insufficient tumor penetration, respectively. Here we developed a sphingolipid-derived PTX nanovesicle (paclitaxome) via covalently conjugating PTX to sphingomyelin, which improved pharmacokinetics and enhanced efficacy in metastatic triple-negative breast cancer and pancreatic cancer female mice and reduced myelosuppression. To bolster tumor penetration and reduce phagocytosis, we engineered a cationization-enabled transcytosis machinery by installing an ultra-pH-sensitive azepane (AZE) probe into paclitaxome and masked nanovesicle surface with a CD47 ‘self’ peptide (CD47p). The resulting CD47p/AZE–paclitaxome synchronized the co-delivery of gemcitabine or carboplatin to boost tumor inhibition and eradicate metastasis in late-stage KPC-Luc pancreatic cancer model and prevent tumor relapse and extend survival in postsurgical 4T1-Luc2 triple-negative breast cancer model in female mice. CD47p/AZE–paclitaxome also outperformed previous promising PTX nanoformulations. Finally, the series of nanoparticle modifications was applied to camptothecin, demonstrating its generalizability.

## Abstract (web summary)

Wang et al. designed sphingomyelin-derived vesicles that deliver paclitaxel to tumor site, improving its therapeutic efficacy and reducing associated toxicities, in mouse models of breast and pancreatic cancers.

## Main

PTX has been an influential and powerful chemotherapeutic drug for treating diverse cancers including breast, pancreatic, ovarian, Kaposi’s sarcoma, esophageal, cervical and non-small-cell lung cancers^1,2^. It interferes with microtubule breakdown during cell division, leading to apoptosis^1^. Nonetheless, due to unfavorable solubility and pharmacokinetics (PK), limited tumor internalization and penetration, and intolerable adverse effects, the potential for PTX’s efficacy was substantially suppressed^2–5^. Therefore, there has been a growing effort to develop innovative PTX formulations, which are able to boost the antitumor index and circumvent these shortcomings.

Despite the tremendous efforts on developing various delivery systems to address the limitations of PTX over the past several decades^3^, the US Food and Drug Administration (FDA) only approved two PTX formulations, Taxol and Abraxane. Taxol employs Cremorphor EL and ethanol to formulate PTX. While Taxol improves water solubility of PTX, its excipients cause severe hypersensitivity^6,7^. For clinical administration of Taxol, intravenous premedication (for example, antihistamines and steroids) is required^8,9^. Abraxane is an albumin-bound PTX nanoparticle. Because of the natural occurring transport capabilities of albumin, Abraxane tends to be safer than Taxol by reducing detrimental adverse effects associated with Taxol^3,10^. Nevertheless, Abraxane does not markedly improve PK and tumor delivery over Taxol, yielding unsatisfactory clinical therapeutic outcome^2,4,11^. Hence, developing safer and more efficacious delivery systems is needed to bolster the effectiveness of PTX therapeutic delivery.

Inspired by the clinical success of liposome-enabled drug delivery, we developed a sphingomyelin (SM)-derived PTX nanotherapeutic vesicle platform. As a naturally existing phospholipid in the membranes of mammalian cells, SM possesses a hydroxyl group that can conjugate with drugs containing a functional group such as PTX. We hypothesized that driven by SM’s amphiphilicity, SM–PTX conjugates will self-assemble into nanovesicles, with PTX securely packaged in a lipid bilayer, which would improve PK and tumor uptake, and diminish systemic toxicities compared to nonconjugated PTX, thereby boosting anticancer efficacy. We demonstrated that SM–PTX did indeed form a liposomal nanovesicle (paclitaxome), which markedly increased the drug-loading capacity (DLC) of PTX and stability compared to using various conventional liposomes to physically encapsulate PTX in a bilayer, and elevated the maximum tolerated dose (MTD) with minimal systemic toxicities compared to Taxol.

While paclitaxome delivered more PTX to tumors than Taxol and Abraxane, the overall tumor accumulation of the drug was still limited. This is consistent with the perception that the nanotherapeutics distribute predominately to the periphery of tumor tissues through leaky vasculature based on the enhanced permeability and retention (EPR) effect^12,13^. To tackle insufficient tumor delivery and penetration, we creatively engineered smart cationization-enabled adsorption-facilitated transcytosis machinery by installing an ultra-pH-sensitive probe, AZE into paclitaxome. This strategy enhanced PTX tumor delivery with deeper penetration; however, even with the PEG coating gold standard, a significant amount of drug was still distributed to healthy tissues (for example, liver, spleen and lungs) where the mononuclear phagocyte system (MPS) is present. CD47 has been proven as a ‘self-marker’ to deter phagocytosis of macrophages via binding to a signal regulatory protein α (SIRPα) that initiates the ‘don’t eat me’ signal to impede phagocytosis^14–16^. To reduce nonspecific delivery, we functionalized the paclitaxome surface with CD47 ‘self’ peptide (CD47p)^17^, which extended the circulation time and reinforced drug delivery to tumors versus paclitaxome, enhancing antitumor efficacy and attenuating drug distribution to healthy tissues.

Among the developed PTX nanoformulations, the chimeric polypeptide-conjugated PTX (CP–PTX) and poly-(γ-l-glutamylglutamine)-conjugated PTX (PGG–PTX) nanoparticles were promising and were superior to Abraxane on PK profile and tumor drug exposure, rendering enhanced efficacy in various tumor models^11,18^. We compared our optimal PTX nanovesicle to these two PTX nanoparticles and found that CD47p/AZE–paclitaxome significantly outperformed CP–PTX and PGG–PTX in PK, tumor uptake and penetration, and efficacy in a pancreatic cancer (PC) mouse model.

Additionally, PTX is combined with gemcitabine (GEM) for treating advanced PC^19–21^ or carboplatin (CBPt) for treating early and late-stage triple-negative breast cancer (TNBC)^22–24^. While encouraging clinical outcomes were achieved from both PTX-based combination therapies, the nonspecific tissue distribution of both drugs yielded detrimental systemic toxicities^22,24,25^, hindering their clinical application. Piggybacked by the effective transcytosis and MPS escaping properties, we posited that CD47p/AZE–paclitaxome could be a safer nanocarrier for efficient co-delivery of GEM or CBPt to tumors. Through systematic anticancer activity screening, the optimal synergistic combination ratios were identified, which were precisely engineered into CD47p/AZE–paclitaxome. CD47p/AZE–paclitaxome not only mitigated the side effects from drug combinations, but also coordinated the co-delivery of GEM or CBPt to fortify the antitumor effects in an advanced KPC-Luc PC model and a clinically relevant postsurgical 4T1-Luc2 TNBC model. Our findings underpin the considerable potential of paclitaxome being either a monotherapy or combination delivery platform for treating diverse cancers, foreboding well for its clinical translation. Notably, the series of nanoparticle functionalizations were successfully adopted to a different anticancer agent (camptothecin) to boost its therapeutic delivery, revealing the broad applicability.

## Results

### Development of paclitaxome nanotherapeutic platform

**Fig. 1:**
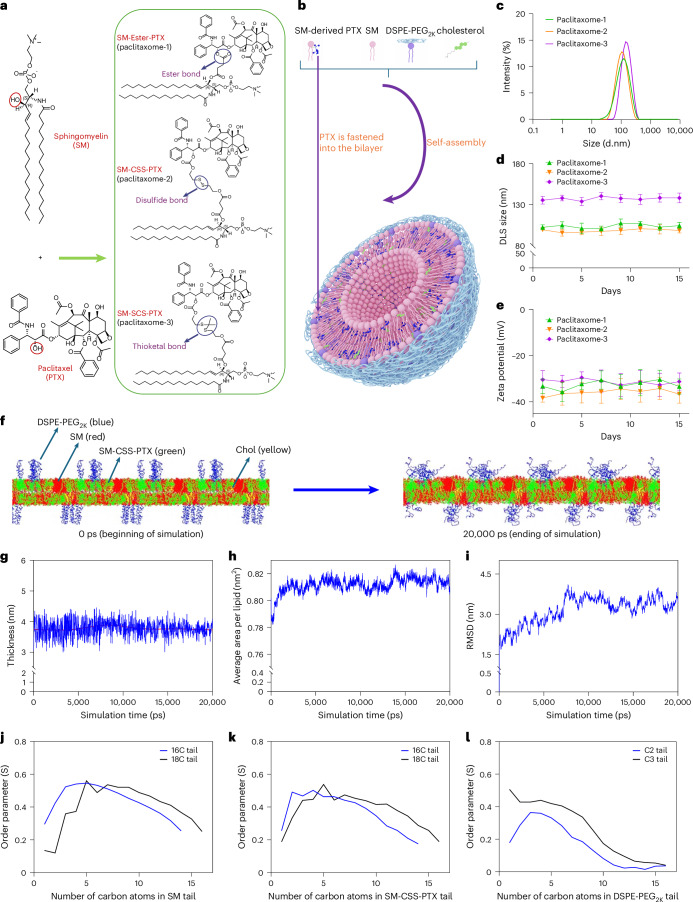
SM-derived PTX self-assembled into paclitaxome. **a**, Chemical structures of SM, PTX and synthesis of SM–PTX conjugates with an ester bond (SM–ester–PTX), a disulfide linkage (SM–CSS–PTX) with elongated linker or thioketal linkage (SM–SCS–PTX) with a elongated linker. **b**, A schematic depicting the self-assembly of SM–PTX into a paclitaxome. **c**, The representative distribution of DLS size via intensity (*n* = 3 independent experiments per group). **d**,**e**, The DLS size presented as intensity (**d**) and zeta potentials (**e**) for paclitaxomes monitored over 2 weeks in 5% dextrose at 4 °C. **f**, The self-assembly of bilayer for paclitaxome-2 from time 0 ps to 20,000 ps through molecular dynamic simulation. The lipids and SM–CSS–PTX were distributed randomly at the beginning of simulation (0 ps), which were rapidly self-assembled into well-organized liposomal bilayer at 20,000 ps. **g**–**i**, The representative thickness of the membrane (**g**), average area per lipid (**h**) and root mean square deviation (RMSD, **i**). During the initial period (0–10,000 ps) of the molecular dynamic simulation, significant fluctuations were observed for the thickness of the membrane, average area per lipid and RMSD. Notably, the bilayer system was able to rapidly adjust to a counterbalance status from 10,000–20,000 ps as evidenced by significantly less fluctuations of the thickness of the membrane, average area per lipid and RMSD, which demonstrated the integrity of and the relatively stable bilayer membrane through self-assembly. **j**–**l**, The representative order parameter of the hydrocarbon tails in phospholipids for SM (**j**), SM–CSS–PTX (**k**) and DSPE-PEG_2K_ (**l**) after molecular dynamic simulation. The hydrocarbon tails of the phospholipids presented positive high order parameters, confirming the good stability of the lipid bilayer (**g**–**l**, *n* = 3 independent experiments per group). Data are presented as mean ± s.d. within **d** and **e** (*n* = 3 independent experiments per group). Source data

**Extended Data Fig. 1:**
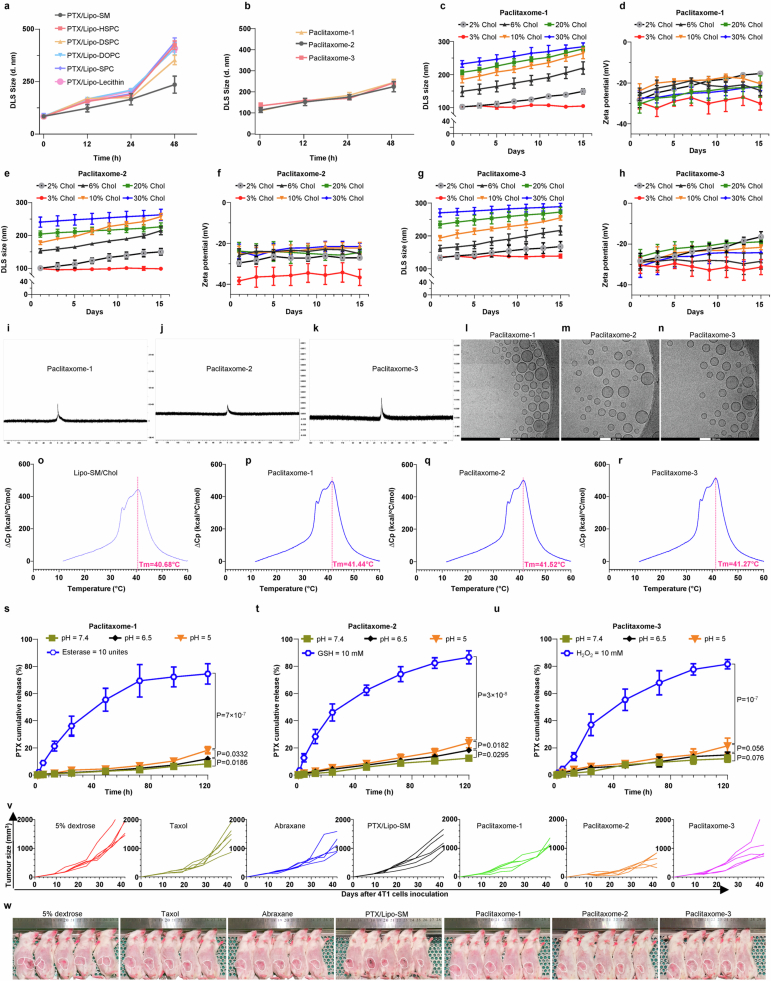
Characteristic properties of physically PTX-laden liposomes and various Paclitaxome. **a**, Monitoring the DLS size of various physically PTX laden liposomes in 4 °C for 48 h. **b**, The DLS size of various Paclitaxomes that comprised by 100% SM–PTX conjugates monitoring over a 48-h period at 4 °C. **c-h**, The DLS Size (**c,e,g**) and zeta potential (**d,f,h**) of various Paclitaxomes composed with various cholesterol molar ratios (2%-30%) monitoring over a 15-day period at 4 °C. **i–k**, ^31^P-NMR spectra of Paclitaxome-1 (**i**), Paclitaxome-2 (**j**) and Paclitaxome-3 (**k**) in D_2_O. **l**–**n**, The panoramic image of Cryo-electron microscopy (cryoEM) for Paclitaxome-1 (**l**), Paclitaxome-2 (**m**) and Paclitaxome-3 (**n**). Scale bar, 100 nm, n = 3 samples per group were performed independently with similar results. **o-r**, Thermograms of Lipo–SM/Chol (**o**), Paclitaxome-1 (**p**), Paclitaxome-2 (**q**) and Paclitaxome-3 (**r**) by differential scanning calorimetry (DSC). MicroCal VP-capillary DSC was used to measure the profiles of thermotropic phase transition. Lipo–SM/Chol consists of 91.92%/3.00%/5.18% molar ratio of SM/Chol/DSPE-PEG_2K_ (SM ratio in Lipo–SM/Chol = “SM + SM–PTX ratio” in Paclitaxome) was set as control. **s-u**, The PTX release kinetics of Paclitaxome-1 (**s**, hydrolase sensitive), Paclitaxome-2 (**t**, GSH sensitive), and Paclitaxome-3 (**u**, ROS sensitive) at various conditions in 37 °C. **v-w**, Individual tumor growth curve (**v**) and photograph of tumor-bearing mouse on day 42 (**w**) from Fig. 2i-j. Data are presented as average ± s.d. within **a-h**, **s-u**. (n = 3 independent experiments per group). One-way ANOVA with Tukey’s multiple comparisons test were used to calculate the exact P values in statistical analyses. Source data

The SM-derived PTX conjugates (SM–PTX) were constructed by linking the 2′-hydroxyl group on PTX with SM’s hydroxyl group. The conjugation was realized by using a sophisticated condensation reaction^26^ (Fig. 1a; see chemical synthesis^27^). Given the unique tumor microenvironment, we designed three distinct linkages (ester, disulfide and thioketal bonds) to bridge SM–PTX conjugates, each of which is sensitive to the high levels of a specific stimulus (hydrolase, glutathione (GSH) as well as reactive oxygen species (ROS), respectively) within tumor sites, enabling the timely release for PTX. The chemical structures and purity of SM–PTX conjugates were confirmed by nuclear magnetic resonance (NMRs), high-performance liquid chromatography (HPLC) and high-resolution mass spectrometry (HRMS) (see chemical synthesis section in Methods). While 100% of SM–PTX self-assembled to liposomes (paclitaxome) in an aqueous medium, adding ancillary lipids (SM, cholesterol (Chol) and DSPE-PEG_2K_) greatly improved monodispersity and formulation stability (Supplementary Tables 1–5, Extended Data Fig. 1 and Supplementary Fig. 1). Moreover, optimized paclitaxome drastically increased DLC (16.2–18.4% versus 0.89%; Fig. 1b–e, Supplementary Tables 1–5 and Extended Data Fig. 1a–u) with significant better stability and uniformity versus physically entrapping PTX in various conventional liposomes using SM, hydrogenated soya phosphatidylcholine (HSPC), 1,2-distearoyl-sn-glycero-3-phosphorylcholine (DSPC), 1,2-dioleoyl-sn-glycero-3-phosphocholine (DOPC), soy phosphatidylcholine (SPC) or lecithin systems (Extended Data Fig. 1a).

### Paclitaxome increases MTD and abrogates systemic toxicities

**Fig. 2:**
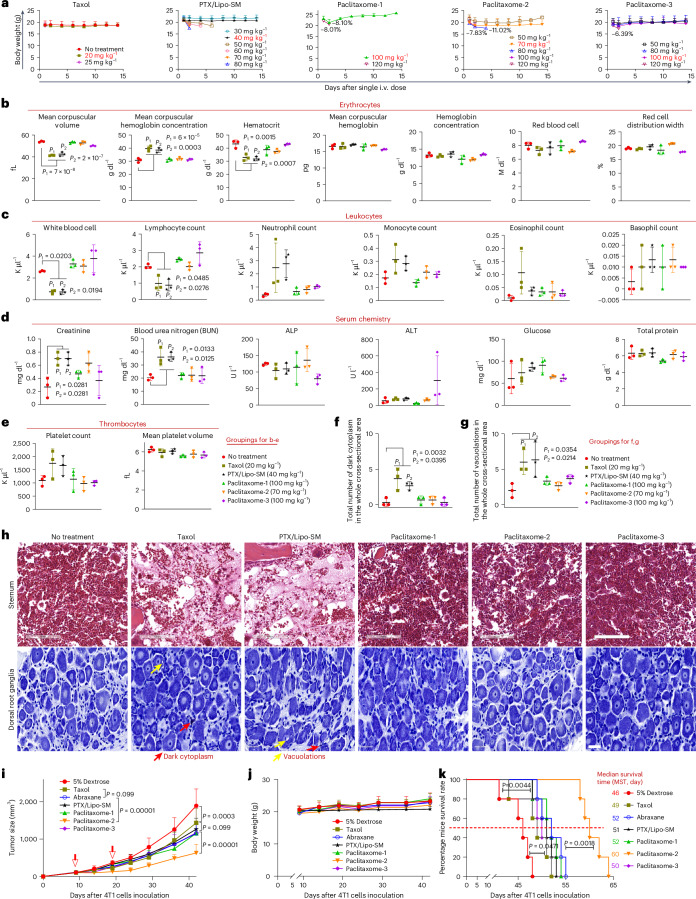
Paclitaxome-2 bolstered the maximum tolerated dose, reduced adverse effects and outperformed Taxol and Abraxane on therapeutic efficacy in an orthotopic 4T1 TNBC model. **a**–**h**, MTD study of Taxol, PTX/Lipo–SM and three paclitaxomes in healthy Balb/c mice (*n* = 3 mice per group). The different PTX formulations were administered intravenously once from caudal vein, the survival and weight of the mouse were measured for 14 days. The maximum dose which did not trigger mortality or more than 15% of weight loss over the entire period was identified as MTD. The mouse weight measuring in MTD study of paclitaxome-1, paclitaxome-2 and paclitaxome-3 with different dose in healthy Balb/c mice (**a**), erythrocytes (**b**), leukocytes (**c**), serum chemistry (**d**), thrombocytes (**e**), the total number of dark cytoplasm (**f**) and vacuolations (**g**) in the whole cross-sectional area of dorsal root ganglia, and representative hematoxylin and eosin (H&E) staining for sternum and toluidine blue staining for dorsal root ganglia (**h**, red arrow indicates dark cytoplasm; yellow arrow indicates vacuolations. Scale bars, 100 μm (top) and 30 µm (bottom) at day 14 for the MTD groups. The whole cross-sectional area was full scanned by the Leica Aperio Versa 200 (Leica), the total number of dark cytoplasm and vacuolations in the panoramic images of dorsal root ganglia were analyzed by Aperio ImageScope software. **i**–**k**, Balb/c mice were surgically inoculated with 1 × 10^5^ 4T1 cells at the fourth mammary fat pad and on day 9 when tumors grew to ~100 mm^3^, animal (*n* = 5 mice per group) administered with equivalent (eq.) 20 mg PTX kg^−1^ on day 9 and 19 via intravenous (i.v.) injection, respectively. Average tumor growth curves (**i**, red arrow, time of drug injection), mean mouse body weight (**j**) and survival Kaplan–Meier plot (**k**). Data are presented as mean ± s.d. within **a**–**g**, **i** and **j**. One-way analysis of variance (ANOVA) with Tukey’s multiple comparisons test were used to calculate the exact *P* values, log-rank Mantel–Cox test was utilized to compare the survival plot in the statistical analyses. Source data

Consistent with the literature^5^, we demonstrated that Taxol MTD was 20 mg PTX kg^−1^, which neither caused mouse death nor led to exceed 15% loss of weight or any noticeable abnormalities within the total monitoring time (Fig. 2a–h and Supplementary Table 6). Notably, paclitaxome increased the MTD of PTX from 20 mg kg^−1^ in Taxol and 40 mg kg^−1^ in PTX/Lipo–SM (physically PTX-laden liposome control, which had better stability versus other PTX/Lipo systems; Extended Data Fig. 1a) to 70–100 mg kg^−1^ in paclitaxomes without eliciting noticeable side effects. In contrast, Taxol and PTX/Lipo–SM at MTD caused severe systemic toxicities, as evidenced by the elevated levels of mean corpuscular hemoglobin concentration, creatinine and blood urea nitrogen, decreased mean corpuscular volume, hematocrit, white blood cells and lymphocytes (Fig. 2b–e). Moreover, we evaluated myelosuppression and neuropathy, two major toxicities associated with PTX, by measuring the integrity of sternums and dorsal root ganglia (Fig. 2f–h). We found that Taxol and PTX/Lipo–SM at MTD severely damaged sternums and dorsal root ganglia tissues, which was manifested by decreased cellularity of bone marrow and hematopoietic cells within sternums and the appearance of cytoplasmic dark inclusions and degenerating neurons in the dorsal root ganglia (Fig. 2f–h); however, these specific toxicities were not seen at paclitaxomes’ MTD (Fig. 2f–h). The decreased adverse effects could be attributed to the controlled drug release mechanism from SM–PTX prodrug conjugates^11,26^. These results confirmed the excellent safety properties in vivo for paclitaxome and its advantages in unleashing the treatment effect of PTX by using higher doses than Taxol.

### Paclitaxome-2 outperforms Taxol and Abraxane on efficacy

To elucidate whether SM–PTXs retain the anticancer activity of PTX, they were intravenously administered into mice bearing 4T1 TNBC tumors in the respective forms of paclitaxome and then compared to Taxol, Abraxane and PTX/Lipo–SM at equivalent (eq.) PTX dose (Fig. 2i–k and Extended Data Fig. 1v,w). Taxol inhibited tumor growth significantly compared to vehicle control, confirming the potent antitumor efficacy of PTX. While Abraxane produced marked tumor reduction, this was not significantly better than Taxol. Notably, all paclitaxomes showed dramatic tumor suppression, particularly in paclitaxome-2 (with a disulfide bond) which outperformed Taxol, Abraxane and PTX/Lipo–SM in delaying tumor development and prolonging mouse survival (Fig. 2i–k).

### In situ cationization triggers transcytosis in tumors

**Fig. 3:**
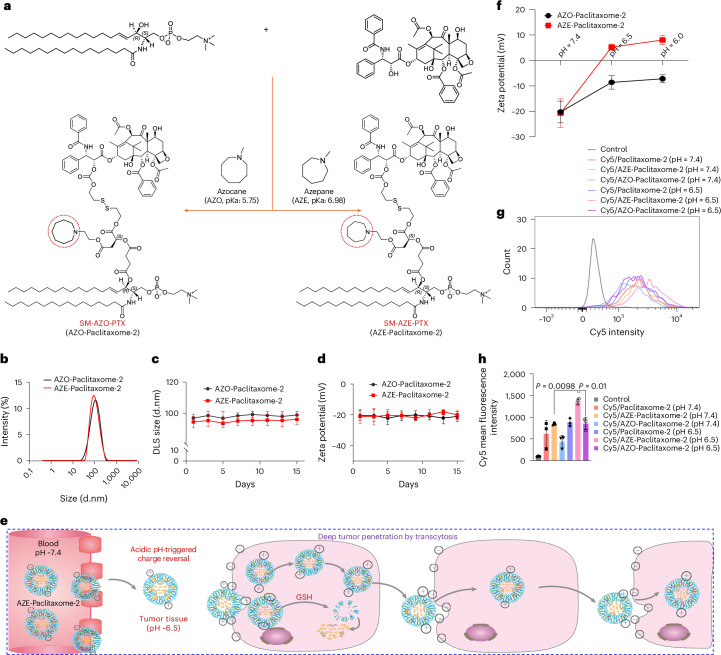
Development of paclitaxome-2 with a built-in ultra-pH-sensitive probe to trigger cationization-induced transcytosis in tumors. **a**, Synthesis of SM–AZO–PTX and SM–AZE–PTX. **b**, the representative distribution of DLS size via intensity for AZO– and AZE–paclitaxome-2 (*n* = 3 independent experiments per group). **c**,**d**, The DLS size presented as intensity (**c**) and Zeta potential (**d**) for AZO– and AZE–paclitaxome-2 monitored over 2 weeks in 5% dextrose at 4 °C. **e**, Schematic of transcytosis in tumors. **f**, Zeta potential at different pH. **g**,**h**, Representative flow cytometry histogram of cellular uptake (**g**, *n* = 3 biological replicates per group) and quantitative determination (**h**, *n* = 3 biological replicates per group) for various DSPE–Cy5-labeled paclitaxome-2 under different pH conditions in 4T1-Luc2 cells. Data are presented as mean ± s.d. within **c**, **d**, **f** and **h** (*n* = 3 independent experiments per group). One-way ANOVA with Tukey’s multiple comparisons test were used to calculate the exact *P* values in the statistical analyses. Source data

**Fig. 4:**
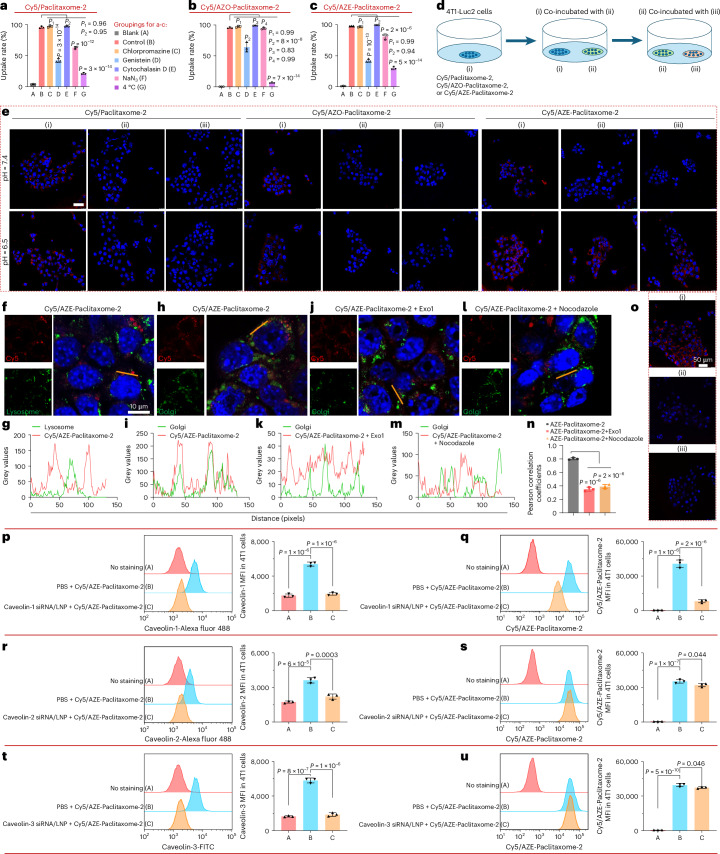
Intracellular uptake of paclitaxome-2 was via caveolae/Golgi trafficking. **a**–**c**, after 0.5 h of pre-incubation with different endocytic pathway inhibitors (EPIs), 4T1-Luc2 cells were treated with various DSPE–Cy5-tagged paclitaxome-2 for 1 h at pH 6.5 under 37 °C or 4 °C with or without EPI for flow cytometry analysis, Chlorpromazine, clathrin-mediated EPI; Genistein, caveolae-meditated EPI; Cytochalasin D, macropinocytosis EPI; NaN_3_, ATP energy generation inhibitor. Quantitative uptake for Cy5/paclitaxome-2 (**a**), Cy5/AZO–paclitaxome-2 (**b**) and Cy5/AZE–paclitaxome-2 (**c**). **d**, Diagram for show the experimental procedures to investigate the transcytosis of various Cy5-labeled paclitaxome-2. 4T1-Luc2 cells on the coverslip (i) were incubated in a medium at pH 6.5 or 7.4 that contained Cy5/paclitaxome-2, Cy5/AZE–paclitaxome-2 or Cy5/AZO–paclitaxome-2. After rinsing, Coverslip (i) was incubated with coverslip (ii), which had cells on it in Cy5-free culture medium. The same procedure was performed again to acquire coverslip (iii) with cells on it. **e**, Confocal imaging of various Cy5/paclitaxome-2 (red) under pH 7.4 and 6.5 (top and bottom). Scale bars, 25 μm. **f**–**m**, Representative colocalization and fluorescence intensity profiles across cells along the selected yellow line with lysosome (**f**,**g**) or Golgi (**h**,**i**) trackers in 4T1-Luc2 cells treated by Cy5/AZE–paclitaxome-2. Exo1 (50 μM, **j**,**k**) or nocodazole (10 μM, **l**,**m**) were preincubated with cells for 60 min. Scale bars, 10 μm, (**f–m** *n* = 3 biological replicates per group). **n**, Pixel intensity of Pearson correlation coefficients between Cy5 and Golgi tracker in the whole image were measured by ImageJ software^28^. **o**, Transcytosis of Cy5/AZE–paclitaxome-2 was blocked after 4T1-Luc2 cells were preincubated with exocytosis inhibitor Exo1. Experiment procedures were same as **d**. Scale bars, 50 μm. **p**–**u**, Cellular uptake of Cy5/AZE–paclitaxome-2 in flow cytometry via silencing the levels of caveolin-1–3 by corresponding siRNA lipid nanoparticles (LNPs) in 4T1-Luc2 cells, respectively. The representative flow cytometry histogram (left) and quantitative analysis (right) of caveolin-1 (**p**), caveolin-2 (**r**), caveolin-3 (**t**) and cellular uptake of Cy5/AZE–paclitaxome-2 via silencing caveolin-1 (**q**), caveolin-2 (**s**) and caveolin-3 (**u**) levels by corresponding caveolin siRNA/LNP, respectively. Data are presented as mean ± s.d. within **a**–**c**, **n** and **p**–**u** (*n* = 3 biological replicates per group). The exact *P* values were measured by one-way ANOVA with Tukey’s multiple comparisons test. Source data

**Extended Data Fig. 2:**
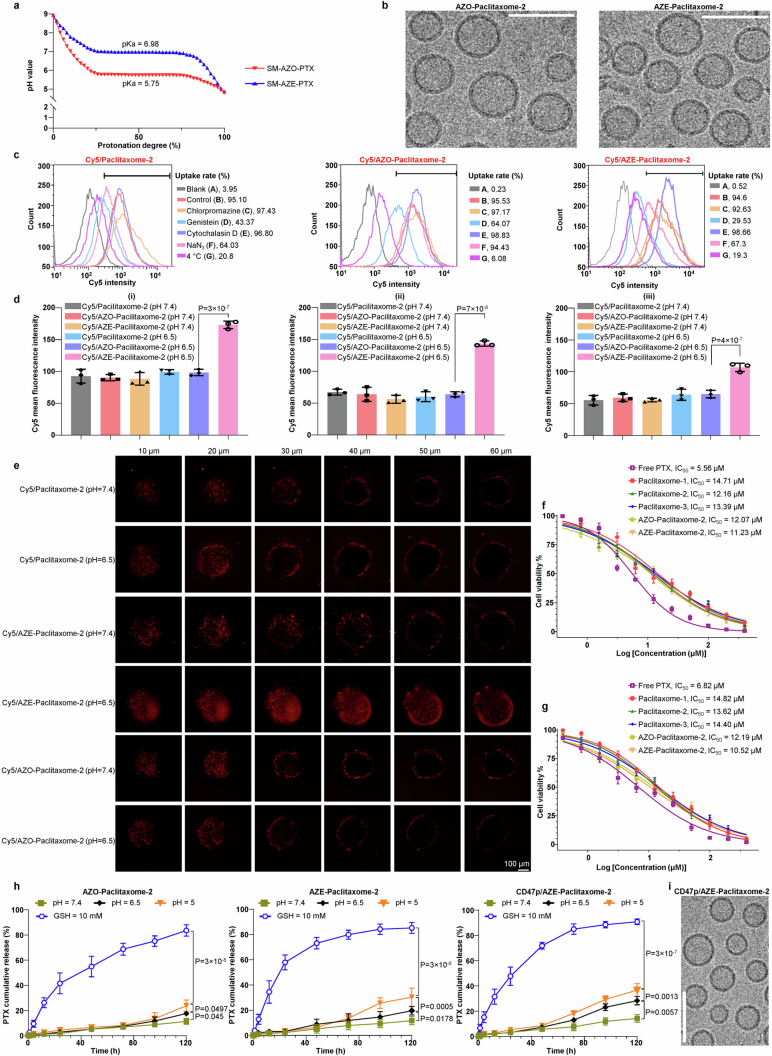
AZE–Paclitaxome-2 fortified penetration in 4T1-Luc2 tumor spheroids. **a**, pH titration of SM–AZO-PTX and SM–AZE-PTX displayed pH-specific buffer effect from pH 4.5 to 9. **b**, Cryo-electron microscopy (cryoEM) of the AZO- and AZE–Paclitaxome-2. Scale bar, 100 nm, n = 3 samples per group were performed independently with similar results. **c**, Representative flow histogram of uptake for Cy5/Paclitaxome-2, Cy5/AZO–Paclitaxome-2 and Cy5/AZE–Paclitaxome-2 from Fig. 4a–c. **d**, The quantified Cy5 mean fluorescence intensity in Fig. 4e. **e**, Confocal imaging observations of Cy5/Paclitaxome-2, Cy5/AZE–Paclitaxome-2, and Cy5/AZO–Paclitaxome-2 penetrating the 4T1-Luc2 tumor spheroids. The well-formed spheroids were seeded in 6-well plate and treated at pH 6.5 or 7.4 in culture medium that contained Cy5/Paclitaxome-2, Cy5/AZE–Paclitaxome-2, or Cy5/AZO–Paclitaxome-2 (DSPE–Cy5, 2 μg/mL) for 4 h. Photographs were taken at every 10 μm from zenith to nadir of the spheroids. Scale bars, 100 μm. **f**,**g**, Cytotoxicity study of various Paclitaxomes in KPC-Luc cells (**f**) and 4T1-Luc2 (**g**) using MTT after incubating cells with different Paclitaxomes for 48 h (n = 3 biological replicates per group). **h**, The PTX release kinetics of AZO–Paclitaxome-2, AZE–Paclitaxome-2, and CD47p/AZE–Paclitaxome-2 at various conditions in 37 °C (n = 3 independent experiments per group). **i**, The representative CryoEM images of CD47p/AZE–Paclitaxome-2. Scale bar: 100 nm, n = 3 samples per group were performed independently with similar results. Data are presented as average ± s.d. within **d** and **f-h**. (n = 3 biological replicates per group). One-way ANOVA with Tukey’s multiple comparisons test were used to calculate the exact P values in statistical analyses. Source data

In situ cationization of nanoparticles via the charge-reversal strategy can induce robust adsorption-facilitated tumor transcytosis with deep tumor penetration potential^28^. To further improve the tumor delivery efficiency, we anchored an ultra-pH-sensitive probe, AZE into the interfacial region of the best SM–PTX, SM–CSS–PTX (Fig. 3a). Azecane (AZE) has a pKa of ~6.98 (Extended Data Fig. 2a)^29^, and when the pH is lower than 6.98 (for example, ~6.5 in tumors), AZE can be rapidly turned cationic via protonation to impart positive charge to nanovesicles. Azocane (AZO; pKa, ~5.75)^29^ was used a negative control probe. We demonstrated that anchoring AZE or AZO into SM–CSS–PTX did not affect the self-assembly of paclitaxome-2, PTX DLC and formulation stability; Instead the negative surface charge decreased after probe incorporation (Supplementary Tables 7 and 8, Fig. 3b–d and Extended Data Fig. 2b). AZE–paclitaxome-2 successfully switched the negative charge (~−20 mV, at pH 7.4) to positive (~5 mV, at pH 6.5), whereas AZO–paclitaxome-2 failed to do so, substantiating the pKa-based ultra-sensitivity of AZE to mild intratumoral pH (Fig. 3e,f). We showed that at pH 7.4, AZE–paclitaxome-2 did not markedly boost the cellular uptake compared to just paclitaxome-2 and AZO–paclitaxome-2. Nevertheless, at pH 6.5, significant improvement of cellular uptake was obtained in AZE–paclitaxome-2, signifying the cationization induced by the mild acidic pH played a crucial role in enhancing AZE–paclitaxome-2 internalization by cells (Fig. 3g,h). Through pretreating 4T1-Luc2 cells with diverse endocytotic pathway inhibitors, we elucidated that the cellular uptake of paclitaxome-2 regardless of the types of pH probe installed was significantly inhibited by 4 °C and/or NaN_3_, unraveling that the internalization was energy-dependent (Fig. 4a–c and Extended Data Fig. 2c). While cytochalasin D (inhibitor of macropinocytosis) and chlorpromazine (inhibitor of clathrin-triggered endocytosis) had no impact on the uptake rate, caveolae-mediated endocytotic inhibitor (genistein) significantly impeded the cellular uptake level, demonstrating that the nanovesicles were ingested by 4T1-Luc2 cells primarily through caveolae-mediated endocytosis. To verify whether AZE–paclitaxome-2 can trigger transcytosis using cationization machinery, we treated cells with DSPE–Cy5-labeled AZE–paclitaxome-2 followed by co-incubation with another two batches of fresh cells (Fig. 4d) as published^28^. Our confocal imaging unveiled that at both pH 6.5 and 7.4, Cy5/paclitaxome-2 and Cy5/AZO–paclitaxome-2 cannot enable transcytosis as manifested by the limited transcellular transport in cells of batches ii and iii (Fig. 4e and Extended Data Fig. 2d). In contrast, at pH 6.5, Cy5/AZE–paclitaxome-2 had strong fluorescence signal in both batches ii and iii cells, suggesting pH-dependent cationization-induced endocytosis and transcytosis. Then we asked what the underlying mechanisms are for the observed transcytosis. To answer this question, first we investigated what intracellular transport pathway was used after internalization. We stained the lysosome and Golgi apparatus and found that our Cy5/AZE–paclitaxome-2 colocalized well with Golgi but not lysosomes, indicating that majority of the Cy5/AZE–paclitaxome-2 was transferred to Golgi after uptake (Fig. 4f–i) and justifying the subsequent extracellular transport by escaping the enzymatic degradation mediated by lysosome. To dive deeper into the role of Golgi involved, we pretreated cells with Exo1 or nocodazole. Nocodazole can disrupt the caveosome-mediated transport to endoplasmic reticulum^30–32^, while Exo1 inhibits intracellular vesicle trafficking between the endoplasmic reticulum and Golgi^28,30,33^. We demonstrated that nocodazole and Exo1 significantly reduced AZE–paclitaxome-2 transport to the Golgi (Fig. 4j–n); and Exo1 also blocked the transcytosis demonstrated by the diminished transcellular delivery (Fig. 4o). To rule out off-target effects, and as the caveolin family consists of three subtypes in vertebrates^34^, we used siRNA targeting various caveolins to silence the level of caveolin 1–3 in 4T1-Luc2 cells (Fig. 4p–u and Supplementary Fig. 2). Of note, while depletion of caveolin-2 and caveolin-3 reduced the cellular uptake of AZE–paclitaxome-2; however, which was more significant by blocking the caveolin-1 (Fig. 4p,q). These data underpinned that AZE–paclitaxome-2 was taken up by cells primarily dependent on caveolin-1 through caveolae-mediated endocytosis. Taken together, we elucidated that AZE–paclitaxome-2 harnessed the distinct caveolae-mediated endocytosis for intracellular trafficking to the Golgi, which facilitated the exocytosis for subsequent effective transcytosis. The cationization-triggered transcytosis for AZE–pacliatxome-2 was further proven in a three-dimensional 4T1-Luc2 tumor spheroid model (Extended Data Fig. 2e).

### CD47 ‘self’ peptide (CD47p) decoration surpasses PEGylation

**Fig. 5:**
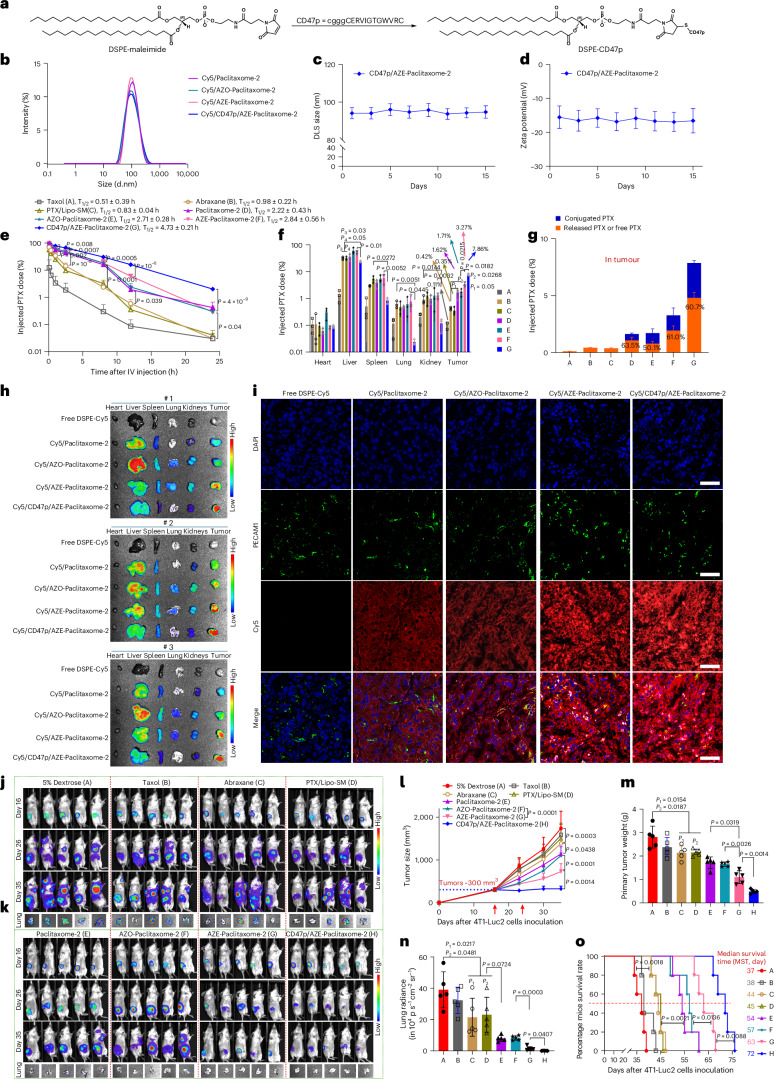
CD47p/AZE–paclitaxome-2 enhanced tumor delivery and bolstered anti-TNBC efficacy with decreased distribution to MPS. **a**, Synthesis of DSPE–CD47p. **b**, The representative DLS size distribution of various Cy5/paclitaxome-2 by intensity. **c**,**d**, DLS size (**c**) and zeta potential (**d**) of CD47p/AZE–paclitaxome-2 monitoring over a 15-day period at 4 °C (*n* = 3 independent experiments per group). **e**–**g**, Blood kinetics (**e**), tissue distribution (**f**) and PTX release rate in tumors (**g**) at 24 h in orthotopic 4T1-Luc2 tumor mouse model (*n* = 3 mice per group, tumors ~300 mm^3^). Mice received a single i.v. injection for Taxol (20 mg PTX kg^−1^, MTD), PTX/Lipo–SM (40 mg PTX kg^−1^, MTD), Abraxane and various paclitaxome-2 (MTD) at 70 mg PTX kg^−1^. **h**, Lago optical ex vivo photograph of various organs at 24 h in orthotopic 4T1-Luc2 tumor mouse model (*n* = 3 mice per group, tumor size ~300 mm^3^) after an i.v. injection with free DSPE–Cy5 and various DSPE–Cy5 tagged paclitaxome-2. **i**, Representative images of extravasation and penetration for various paclitaxome-2 within the tumors from **h** (*n* = 3 tumors per group). 4,6-diamidino-2-phenylindole (DAPI)-stained nuclei (blue). PECAM1 primary antibody and Alexa Fluor 488 secondary antibody labeled blood vessels (green). Scale bars, 50 μm. **j**–**o**, antitumor efficacy in orthotopic 4T1-Luc2 tumor-bearing mice with metastasis (*n* = 5 mice per group, primary tumors: ~300 mm^3^). **j**, On day 16, 26 and 35, mice bioluminescence imaging (BLI) were measured by using Lago spectral imaging. **k**, BLI of lung in mice ex vivo to show metastasis on day 35. **l**, mean tumor volume determined by a caliper (red arrow indicates the time of drug injection). **m**,**n**, primary tumor weight (**m**) and quantification of BLI for lung metastasis (**n**) from **l** via Lago optical imager. **o**, Kaplan–Meier survival plot from a separate investigation in which mice (*n* = 5 mice per group, primary tumors, ~300 mm^3^) received the same treatments as **l**. Data are presented as mean ± s.d. (**c**–**g**,**l**–**n**). One-way ANOVA with Tukey’s multiple comparisons test were used to calculate the exact *P* values in **e**,**f**,**l**–**n**, log-rank Mantel–Cox test was utilized to compare the survival plot in **o**. Source data

**Extended Data Fig. 3:**
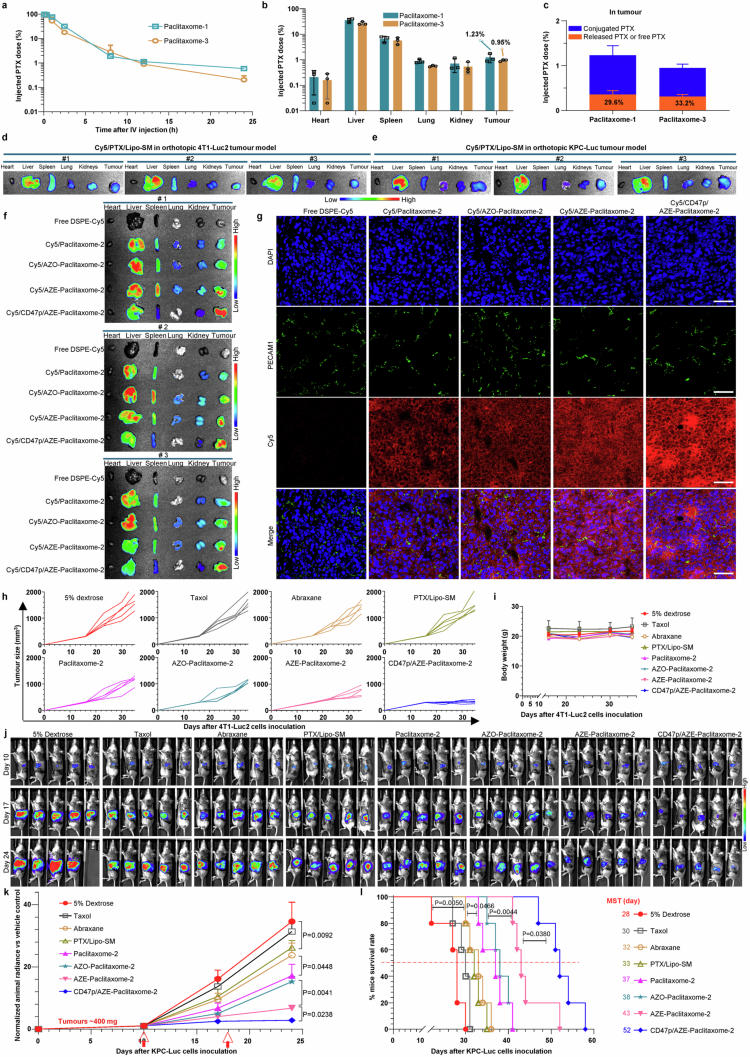
CD47p/AZE-Paclitaxome-2 further enhanced drug delivery and antitumor efficacy with longest survival benefit in advanced KPC-Luc PC with metastasis. **a-c**, Blood kinetics (**a**), tissue distribution (**b**) and PTX release rate in tumors (**c**) at 24 h in orthotopic 4T1-Luc2 tumor mouse model (n = 3 mice per group, tumors: ~300 mm^3^). Animals were intravenously administered once with Paclitaxome-1 or Paclitaxome-3 at 70 mg PTX/kg, respectively. **d**,**e**, Lago optical ex vivo photographs of various organs 24 h in orthotopic 4T1-Luc2 tumor mice (**d**, n = 3 mice per group, tumors ~300 mm^3^) and orthotopic KPC-Luc tumor mice (**e**, n = 3 mice per group, tumors ~400 mg) after i.v. injection once with DSPE–Cy5-labeled PTX/Lipo–SM. **f**, Lago optical ex vivo photographs of various organs 24 h in orthotopic KPC-Luc mouse model (n = 3 mice per group, tumors ~400 mg) after i.v. injection once with DSPE–Cy5, DSPE–Cy5-labeled various Paclitaxome-2. **g**, Representative images of extravasation and penetration for various Paclitaxome-2 within tumors from **f** (n = 3 tumous per group). DAPI stained nuclei (blue). PECAM1 primary antibody and Alexa Fluor 488™ secondary antibody labeled blood vessels (green). Scale bars, 50 μm. **h**,**i**, Individual tumor volume determined by a caliper (**h**) and average body weight (**i**) from Fig. 5l. **j–l**, Antitumor effect in orthotopic PC mice with metastasis. B6129SF1/J mice were surgically inoculated with 2 × 10^6^ cells into their pancreas (n = 5 mice per group). When main tumors grew to ~400 mg and had discernible metastasis on day 10, animals were intravenously administered with the same dose as Fig. 5l. **j**, BLI of mice by Lago imaging on day 10, 17 and 24. On day 23, one mouse died from 5% dextrose. **k**, BLI of entire tumor burden in animal by normalization (red arrow: time of drug injection). **l**, Kaplan–Meier survival curves. Data are presented as average ± s.d. within **a**-**c**, **i** and **k**. One-way ANOVA with Tukey’s multiple comparisons test were used to calculate the exact P values, log-rank Mantel–Cox test was utilized to compare the survival plot in the statistical analyses. Source data

Based on the promising CD47 ‘self’ masking property on therapeutic delivery, we were interested in answering a critical question as to how CD47p neutralization impacts the PK and biodistribution compared to gold standard PEGylation on nanocarriers. To this end, CD47p was conjugated with DSPE–maleimide via Michael addition (Fig. 5a)^15,17^. The DSPE–CD47p was purified by dialysis and confirmed by NMR. By systematic screening and optimization, we demonstrated that coating of ~5 molar% DSPE–CD47p gave the most consistent physicochemical properties as AZE–paclitaxome-2 (Supplementary Table 9 and Fig. 5b–d). The superior anticancer efficacy of paclitaxome-2 over paclitaxome-1, paclitaxome-3, Taxol, Abraxane and PTX/Lipo–SM is likely due to the extended circulation time and enhanced tumor delivery efficiency (Fig. 5e–h and Extended Data Fig. 3a–g). While paclitaxome-2 increased tumor delivery compared to Taxol and Abraxane by elongating the circulation time based on EPR effects, the overall drug uptake in tumors was mediocre (~1.6%) with poor penetration and distribution within tumors (Fig. 5e–i). Nevertheless, AZE–paclitaxome-2 proved that it markedly enhanced drug delivery to tumors with better tumor infiltration and distribution versus Taxol, Abraxane, PTX/Lipo–SM, paclitaxome-2 and AZO–paclitaxome-2 in both 4T1-Luc2 TNBC and KPC-Luc PC tumor models (Fig. 5e–i and Extended Data Fig. 3d–g). Compared to AZE–paclitaxome-2 that has ~5 molar% DSPE-PEG_2K_, this CD47p coating strategy significantly prolonged circulation time (by 1.67-fold) and delivered more drug to tumors (by 2.40-fold), while reducing the distribution to liver, spleen, lung and kidneys due to the MPS avoidance (Fig. 5e–i, Extended Data Fig. 3a–g and Supplementary Tables 10–12).

In mice bearing late-stage orthotopic 4T1-Luc2 TNBC tumors (~300 mm^3^), we showed that Taxol at MTD did not produce significant tumor inhibition or prevent metastasis into the lungs (Fig. 5j–o). Abraxane improved efficacy due to the higher dose allowed; however, paclitaxome-2 beat Taxol, Abraxane and PTX/Lipo–SM by eliciting markedly more tumor reduction. AZE–paclitaxome-2 enhanced anti-TNBC effects as evidenced by further delayed tumor growth and reduced metastasis, which was not seen in AZO–paclitaxome-2. Furthermore, CD47p/AZE–paclitaxome-2 almost completely curbed primary tumor development and facilitated metastasis remission (Fig. 5j–o). In addition, paclitaxome-2 performed better than Taxol, Abraxane and PTX/Lipo–SM on prolonging mouse survival time, particularly in formulation with transcytosis and ‘self’ peptide masking mechanisms (Fig. 5o). No significant loss of body weight was detected for any treatments (Extended Data Fig. 3h–i). As PTX is also used for PC, we investigated whether paclitaxome-2 could also improve efficacy in this cancer model. We revealed that paclitaxome-2 outperformed Taxol, Abraxane and PTX/Lipo–SM in curbing tumor development and extending survival time in an advanced orthotopic KPC-Luc PC model, especially in CD47p/AZE–paclitaxome-2 (Extended Data Fig. 3j–l).

**Extended Data Fig. 4:**
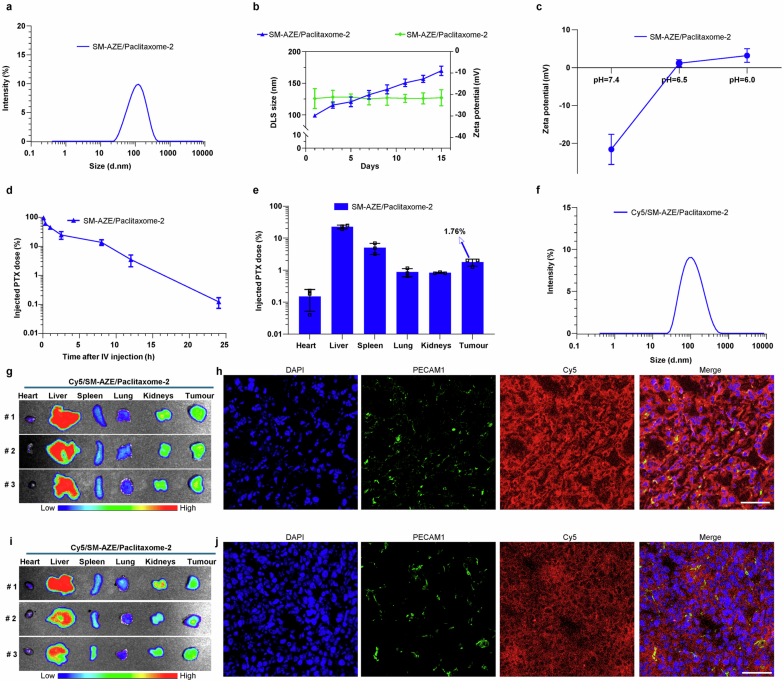
The pharmacokinetics and biodistribution study of SM–AZE/Paclitaxome-2. **a**, Distribution of DLS size via intensity of SM–AZE/Paclitaxome-2. **b**, Monitoring of DLS size, zeta potential for SM–AZE/Paclitaxome-2 at 4 °C for 14 days (n = 3 independent experiments per group). **c**, Zeta potential of SM–AZE/Paclitaxome-2 at different pH (n = 3 independent experiments per group). **d**,**e**, Blood kinetics (**d**) and tissue distribution (**e**) at 24 h in orthotopic 4T1-Luc2 tumor mouse model (n = 3 mice per group, tumors ~300 mm^3^). **f**, DLS size distribution by intensity of Cy5-DSPE labled SM–AZE/Paclitaxome-2. Animals were intravenously administered once with SM–AZE/Paclitaxome-2 at 70 mg PTX/kg. HPLC was used to determine the drug levels in primary organs and plasma. **g**, Lago optical ex vivo photographs of various organs at 24 h in orthotopic 4T1-Luc2 tumor mouse model (n = 3 mice per group, tumors: ~300 mm^3^) after an i.v. injection with DSPE–Cy5-labeled SM–AZE/Paclitaxome-2 at 70 mg PTX/kg. **h**, Representative images of extravasation and penetration for SM–AZE/Paclitaxome-2 within the tumors from **g** using CLSM (n = 3 tumous per group). **i**, Lago optical ex vivo photographs of various organs at 24 h in orthotopic KPC-Luc tumor mice (n = 3 mice per group, tumors: ~400 mg) after an i.v. injection with DSPE–Cy5-labeled SM–AZE/Paclitaxome-2 at 70 mg PTX/kg. **j**, Representative images of extravasation and penetration for SM–AZE/Paclitaxome-2 within the tumors from **i** using CLSM (n = 3 tumors per group). DAPI (blue) was utilized to stain cell nuclei. Primary antibody for PECAM1 first and then Alexa Fluor^TM^ 488 detection antibody (green) were used to label the vessels of blood. Scale bars, 50 μm. Data are presented as average ± s.d. within **b-e**. Source data

**Extended Data Fig. 5:**
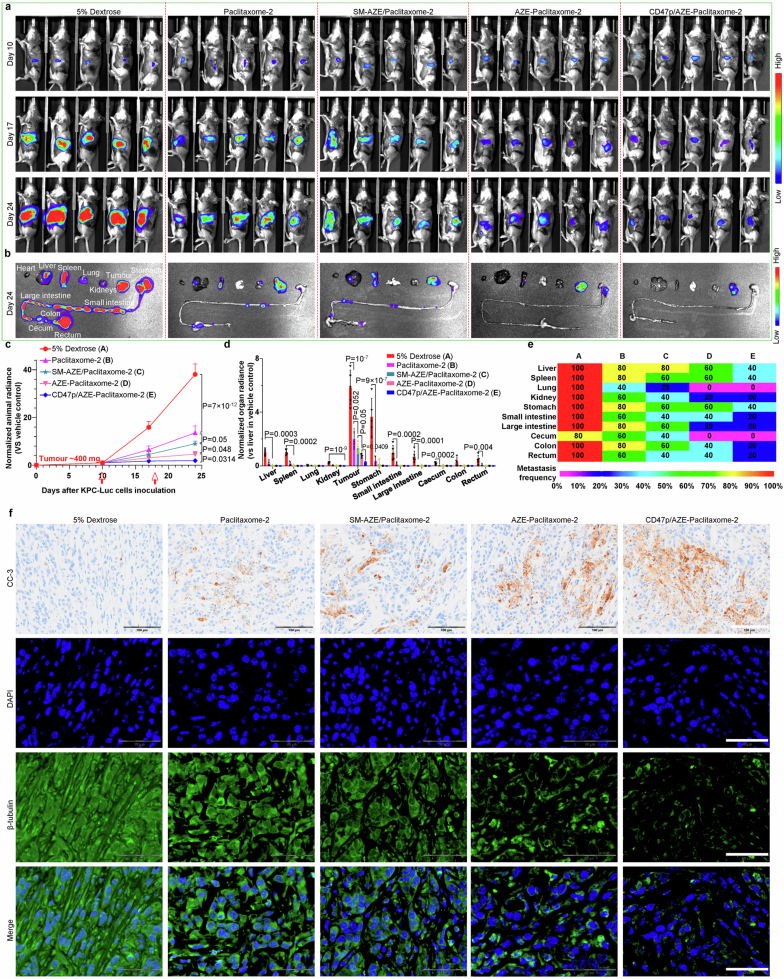
Therapeutic efficacy of SM-AZE/Paclitaxome-2 and CD47p/AZE-Paclitaxome-2 in metastatic orthotopic KPC-Luc PC mouse model. **a-e**, B6129SF1/J mice were surgically inoculated with 2 × 10^6^ KPC-Luc cells into their pancreas (n = 5 mice per group). When the main tumor grew to ~400 mg and had discernible metastasis on day 10, the animals were administered intravenously with Paclitaxome-2 (70 mg PTX/kg), SM-AZE/Paclitaxome-2 (70 mg PTX/kg), AZE-Paclitaxome-2 (70 mg PTX/kg), CD47p/AZE-Paclitaxome-2 (70 mg PTX/kg) on day 10 and 18. **a**, BLI of mice by Lago imaging on day 10, 17 and 24. **b**, On day 24, representative *ex vivo* BLI of different organs. **c**, The BLI of entire tumor burden in animal by normalization (red arrow: time of drug injection). BLI of different organs by normalization (**d**) and metastatic frequency heatmap (**e**) on day 24. **f**, Representative intratumoral IHC staining for CC-3, (scale bar: 100 μm), and immunofluorescence staining for β-tubulin (scale bar: 50 μm). Data are presented as average ± s.d. within **c,d** (n = 5 mice per group). One-way ANOVA with Tukey’s multiple comparisons test were used to calculate the exact P values in the statistical analyses. Source data

We also synthesized SM–AZE in which the AZE is directly anchored to the SM (see chemical synthesis in ref. ^27^), while SM–AZE/paclitaxome-2 entailed the surface charge-reversal property, its stability was much poorer than that of AZE–paclitaxome-2 (Supplementary Table 13, Extended Data Fig. 4). This may be attributed to the mismatched structure between SM–AZE and SM–CSS–PTX, which jeopardized the integrity of the symmetric bilayer by adulteration. PK and efficacy studies unraveled that SM–AZE/paclitaxome-2 decreased the tumor delivery and antitumor effects versus AZE–paclitaxome-2 (Supplementary Tables 14 and 15 and Extended Data Figs. 4 and 5), substantiating the benefit of using a charge-reversal moiety in nanocarrier for therapeutic delivery.

**Extended Data Fig. 6:**
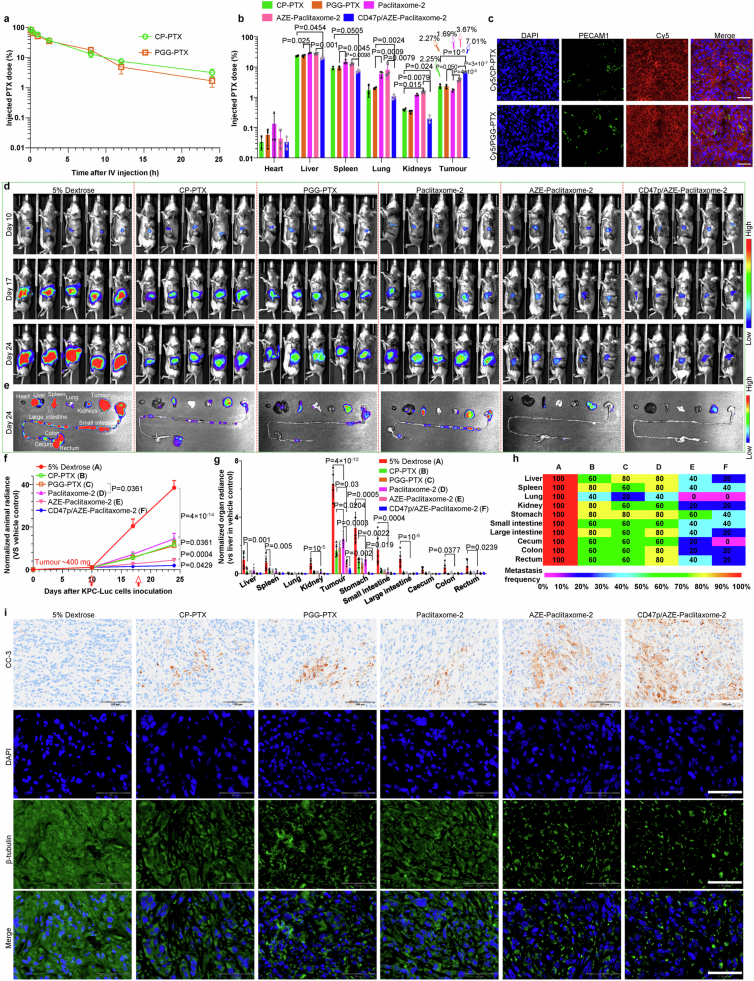
CD47p/AZE-Paclitaxome-2 outperformed CP–PTX and PGG–PTX in drug delivery and antitumor efficacy in metastatic orthotopic KPC-Luc PC mouse model. **a-c**, Blood kinetics (**a**), tissue distribution (**b**) at 24 h in orthotopic KPC-Luc tumor mouse model (n = 3 mice per group, tumors ~400 mg). Animals were intravenously administered once with CP–PTX, PGG–PTX, Paclitaxome-2, AZE-Paclitaxome-2 and CD47p/AZE-Paclitaxome-2 at 70 mg PTX/kg, respectively. HPLC was used to determine the drug levels in primary organs and plasma. **c**, Representative images of extravasation and penetration for Cy5/CP–PTX and Cy5/PGG–PTX within the tumors by using CLSM (n = 3 tumors per group). DSPE–Cy5 were used to label CP–PTX and PGG–PTX, twenty-four hours after injecting Cy5/CP–PTX and Cy5/PGG–PTX (red) at e.q. 0.84 mg DSPE–Cy5 and 70 mg PTX/kg (n = 3 mice per group, tumors: ~400 mg), CLSM of slices of KPC-Luc tumor was performed. DAPI (blue) was utilized to stain cell nuclei. Primary antibody for PECAM1 first and then Alexa Fluor^TM^ 488 detection antibody (green) were used to label the vessels of blood. Scale bars, 50 μm. **d-i**, B6129SF1/J mice were surgically inoculated with 2 × 10^6^ KPC-Luc cells into their pancreas (n = 5 mice per group). When the main tumor grew to ~400 mg and had discernible metastasis on day 10, the animals were administered intravenously with CP–PTX, PGG–PTX, Paclitaxome-2, AZE-Paclitaxome-2, CD47p/AZE-Paclitaxome-2, at eq. 70 mg PTX/kg on day 10 and 18. **d**, BLI of mice by Lago imaging on day 10, 17 and 24. **e**, On day 24, representative *ex vivo* BLI for different organs. **f**, The BLI of entire tumor burden in animal by normalization (red arrow: time of drug injection). BLI of different organs by normalization (**g**) and metastatic frequency heatmap (**h**) on day 24. **i**, Representative intratumoral IHC staining for CC-3, (scale bar: 100 μm), and immunofluorescence staining for β-tubulin (scale bar: 50 μm). Data are presented as average ± s.d. within **a**, **b**, **f** and **g**. One-way ANOVA with Tukey’s multiple comparisons test were used to calculate the exact P values in the statistical analyses. Source data

Various formulations have been previously developed to improve PTX therapeutic delivery^3^. Examples include the recombinant chimeric polypeptides (CPs) conjugated PTX (CP–PTX)^11^ and the poly-(γ-l-glutamylglutamine) conjugated PTX (PGG–PTX)^18^, both of which outperformed Abraxane on therapeutic delivery of PTX. To dive deeper into the therapeutic potential of our optimal CD47p/AZE–paclitaxome-2, we compared it with these two PTX nanoformulations, CP–PTX and PGG–PTX on tumor penetration, MPS avoidance and anticancer efficacy (Extended Data Fig. 6a–c, Supplementary Table 16 and chemical synthesis in ref. ^27^). Notably, CD47p/AZE–paclitaxome-2 performed markedly better than CP–PTX and PGG–PTX nanoparticles on circulation time, extravasation, intratumoral diffusion, the ability to avoid MPS clearance and therapeutic effect by achieving significant greater tumor suppression and metastasis remission. (Fig. 5 and Extended Data Fig. 6). These findings further strengthened the promise of our nanovesicle delivery platform for PTX.

**Fig. 6:**
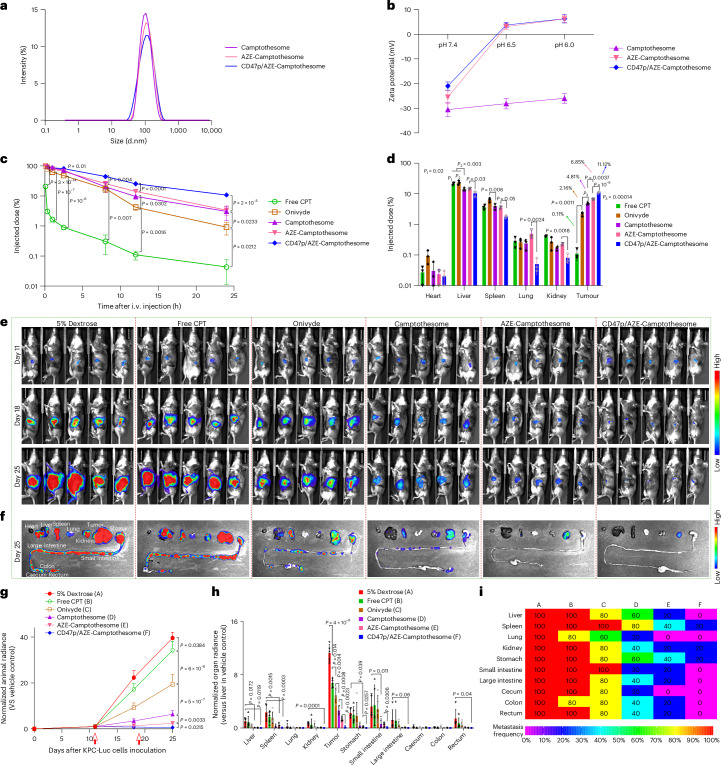
Therapeutic efficacy of CD47p/AZE–camptothesome in metastatic orthotopic KPC-Luc PC mouse model. **a**,**b**, Distribution of DLS size via intensity (**a**) and Zeta potential (**b**) for various camptothesome at different pH (*n* = 3 independent experiments per group). **c**,**d**, Blood kinetics (**c**) and tissue distribution (**d**) at 24 h in orthotopic KPC-Luc tumor mouse model (*n* = 3 mice per group, tumors ~400 mg). Animals were intravenously administered once with MTD dose of free CPT (5 mg kg^−1^), onivyde (33.6 mg Irinotecan kg^−1^) and various camptothesome at equivalent 20 mg CPT kg^−1^, respectively. HPLC was used to determine drug levels in primary organs and plasma. **e**–**i**, Antitumor effect in orthotopic KPC-Luc PC mice with metastasis. B6129SF1/J mice were surgically inoculated with 2 × 10^6^ KPC-Luc cells into their pancreas (*n* = 5 mice per group). When the main tumor grew to ~400 mg and had discernible metastasis on day 11, animals were i.v. administered with MTD of free CPT (5 mg kg^−1^), Onivyde (33.6 mg Irinotecan kg^−1^) and various camptothesome at equivalent 20 mg CPT kg^−1^ on day 11 and 19. **e**, The BLI of entire tumor burden in animal by normalization on day 11, 18 and 25. **f**, On day 25, representative ex vivo BLI for different organs. **g**, The BLI of entire tumor burden in animal by normalization (red arrow shows time of drug injection). BLI of different organs by normalization (**h**) and metastatic frequency heatmap (**i**) on day 25. Data are presented as mean ± s.d. within **b**–**d**,**g**,**h**. One-way ANOVA with Tukey’s multiple comparisons test were used to calculate the exact *P* values in the statistical analyses. Source data

To decipher whether this series of nanoparticle modification strategies ‘SM conjugation + AZE charge reversal + CD47p self-masking’ can be successfully adopted to other poorly soluble drug classes, we implemented same modifications to a different but potent hydrophobic anticancer agent, camptothecin (CPT; topoisomerase 1 inhibitor). SM–AZE–CPT was synthesized and verified by NMRs and HRMS (see chemical synthesis in ref. ^27^). SM–CPT (bridged by a disulfide bond) was synthesized as a control following our published method and formed nanovesicles (camptothesome) consistent with our previous work^26^. Introducing the AZE motif into SM–CPT or decorating the surface of AZE–camptothesome with CD47p did not markedly affect the nanoparticle physicochemical properties (Fig. 6a and Supplementary Table 17). AZE–camptothesome and CD47p/AZE–camptothesome successfully switched the negative charge (~−25 mV and ~−23 mV) in pH 7.4 to positive (~5 mV) in pH 6.5, whereas camptothesome failed to do so (Fig. 6b), corroborating this pKa-based ultra-sensitivity of AZE under mild acidic intratumoral pH can be applied to other drugs such as CPT. We unveiled that AZE–camptothesome markedly enhanced the intratumoral drug delivery compared to camptothesome and coating CD47p onto AZE–camptothesome significantly prolonged half-life and further boosted drug delivery to tumors with less distribution to liver, spleen, lung and kidneys (Fig. 6c,d and Supplementary Table 18). Furthermore, AZE–camptothesome outperformed free CPT, onivyde and camptothesome on therapeutic effects in advanced KPC-Luc PC model, particularly with functionalization of CD47p (Fig. 6e–i). These findings substantiated the generalizability of the ‘SM + AZE + CD47p’ modifications to other drug classes and can serve as general principles to augment drug delivery to tumors for enhanced therapeutic efficacy.

### Co-delivery of GEM for synergistic combination PC therapy

**Fig. 7:**
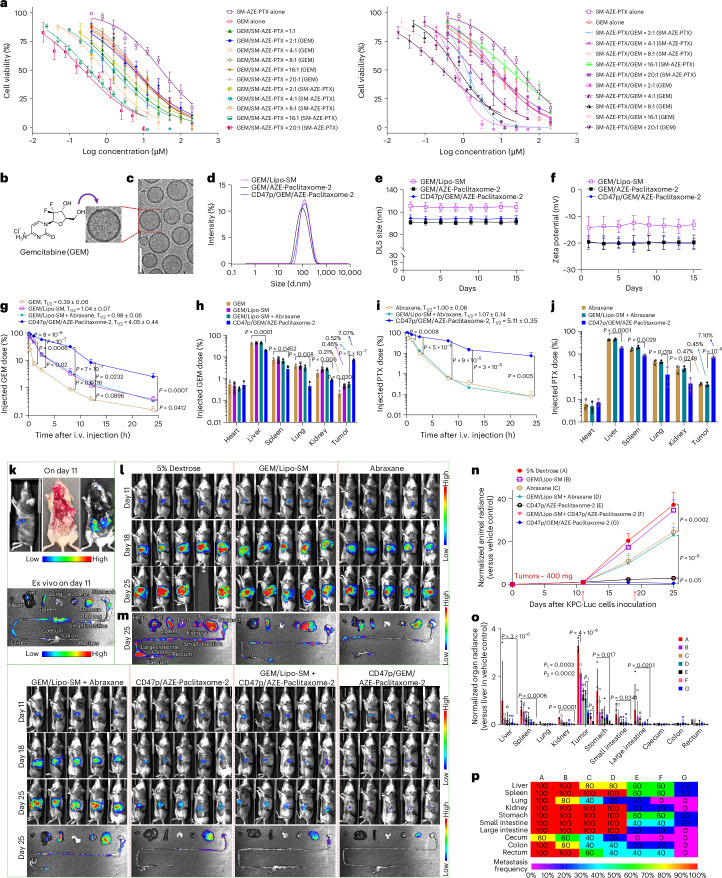
GEM/AZE–paclitaxome-2 synergistically boosted the antitumor efficacy in advanced KPC-Luc pancreas cancer mouse model with metastasis. **a**, Drug CI of various molar ratios of GEM and SM–AZE–PTX in KPC-Luc cells by cytotoxicity test (*n* = 3 biological replicates per group). **b**,**c**, Schematic depicting the preparation (**b**) and cryoEM (**c**) of CD47p/GEM/AZE–paclitaxome-2, *n* = 3 samples per group were performed independently with similar results. **d**–**f**, Representative distribution of DLS size via intensity (**d**) and DLS size (**e**) and zeta potential (**f**) monitoring over a 15-day period at 4 °C of GEM/Lipo–SM, GEM/AZE–paclitaxome-2 and CD47p/GEM/AZE–paclitaxome-2 (*n* = 3 independent experiments per group). **g**–**j**, Blood kinetics (**g**,**i**) and tissue distribution (**h**,**j**) at 24 h in orthotopic KPC-Luc tumor mouse model (*n* = 3 mice per group, tumors ~400 mg) after an i.v. administration of free GEM, GEM/Lipo–SM, GEM/Lipo–SM + Abraxane or CD47p/GEM/AZE–paclitaxome-2 at 12.3 mg GEM kg^−1^ and 70 mg PTX kg^−1^. **k**–**p**, Antitumor effect in orthotopic PC mouse model with metastasis. B6129SF1/J mice were surgically inoculated with 2 × 10^6^ cells into the pancreas (*n* = 5 mice per group). When the main tumor grew to ~400 mg with palpable metastasis (**k**), the animals were intravenously administered with GEM/Lipo–SM (12.3 mg kg^−1^), Abraxane (70 mg PTX kg^−1^), GEM/Lipo–SM + Abraxane (12.3 mg GEM kg^−1^, 70 mg PTX kg^−1^), CD47p/AZE–paclitaxome-2 (70 mg PTX kg^−1^), GEM/Lipo–SM + CD47p/AZE–paclitaxome-2 or CD47p/GEM/AZE–paclitaxome-2 at (MTD; Extended Data Fig. 7a) 12.3 mg GEM kg^−1^ and 70 mg PTX kg^−1^ (SM–AZE–PTX/GEM molar ratio of 2:1) on day 11 and 19. **l**, BLI of mice by Lago imaging from day 11, 18 to 25. On day 24, one mouse died from 5% dextrose group. **m**, On day 25, representative ex vivo BLI of different organs. **n**, The BLI of entire tumor burden in animal by normalization (red arrow indicates the time of drug injection). **o**,**p**, BLI of different organs by normalization (**o**) and metastatic frequency heatmap (**p**) on day 25. Data are presented as mean ± s.d. within **a**,**e**–**j**,**n**,**o**. One-way ANOVA with Tukey’s multiple comparisons test were used to calculate the exact *P* values in the statistical analyses Source data

**Extended Data Fig. 7:**
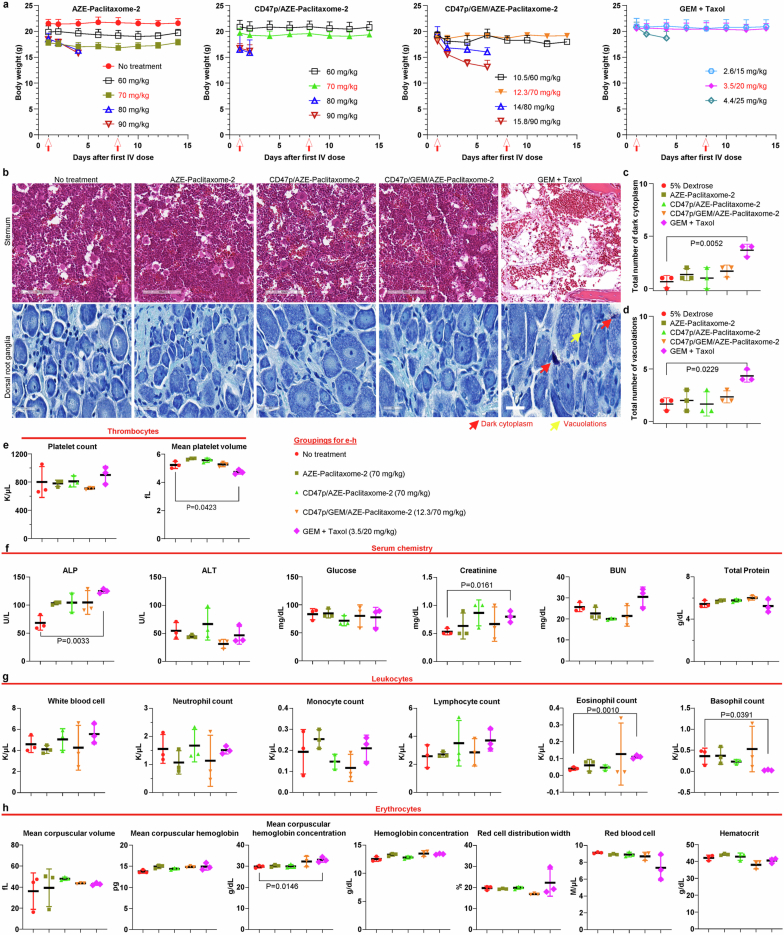
CD47p/GEM/AZE-Paclitaxome-2 mitigated systemic toxicities. **a**, The measurment of body weight in mice from the MTD evaluation of AZE-Paclitaxome-2, CD47p/AZE-Paclitaxome-2, CD47p/GEM/AZE-Paclitaxome-2 (molar ratio SM-AZE-PTX/GEM = 2/1) and GEM + Taxol (molar ratio PTX/GEM = 2/1) in healthy B6129SF1/J mice (n = 3 mice per group) following i.v. injection via caudal vein on day 1 and 8 (red arrow: time of drug injection). The survival and weight of the mouse were assessed for 14 days. The MTD dose were highlighted in red color. **b-h**, representative H&E staining for sternum and toluidine blue staining for dorsal root ganglia (**b**, red arrow, dark cytoplasm; yellow arrow, vacuolations, Scale bar = 100 and 30 µm in the upper and lower panel, respectively, n = 3 mice per group), the total counting of dark cytoplasm (**c**) and vacuolations (**d**) in the whole cross-sectional area of dorsal root ganglia, thrombocytes (**e**), serum chemistry (**f**), leukocytes (**g**) and erythrocytes (**h**) examination on day 14 from MTD. Data are presented as average ± s.d. within **a** and **c-h** (n = 3 mice per group). One-way ANOVA with Tukey’s multiple comparisons test were used to calculate the exact P values in the statistical analyses. Source data

**Extended Data Fig. 8:**
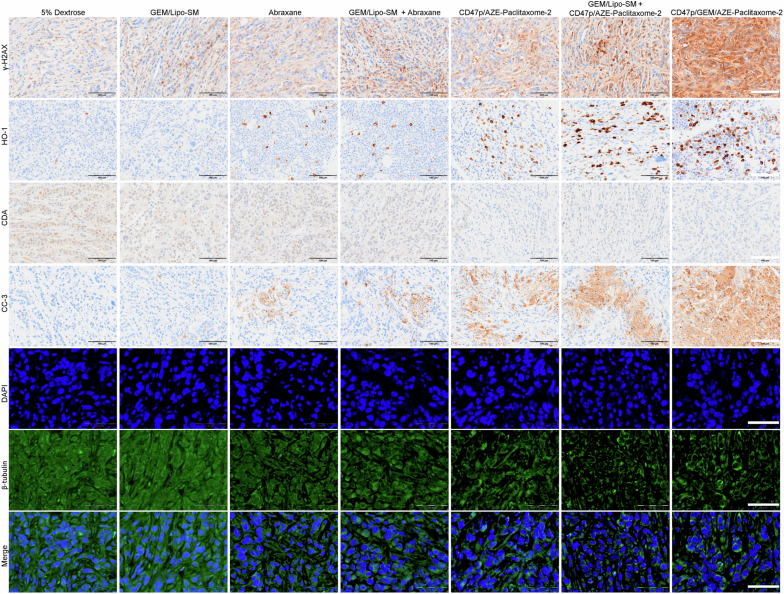
The histopathological study of orthotopic KPC-Luc tumors after being treated with CD47p/GEM/AZE-Paclitaxome-2. Representative IHC staining for intratumoral γ-H2AX, HO-1, CDA, CC-3 (scale bar: 100 μm) and immunofluorescence staining for β-tubulin (scale bar: 50 μm) in orthotopic KPC-Luc tumors from Fig. 7n. (n = 5 tumors per group). Source data

Apart from being a monotherapy, PTX is often co-used with GEM for treating advanced PC^19–21^. Enhanced efficacy was observed using combination therapy of GEM + PTX, but they aggravated adverse effects^21^. Thus, the clinical applications of this combination regimen have been greatly impeded. With effective transcytosis and MPS escaping machinery, we proposed to use CD47p/AZE–paclitaxome-2 to co-deliver GEM to tumors and reduce the nonspecific tissue distribution to improve the combination therapeutic index. Through comprehensive in vitro anticancer activity screening, we pinpointed the optimal synergistic drug molar ratios (2/1, SM–AZE–PTX/GEM) based on the lower combination index (CI; Fig. 7a and Supplementary Table 19). Using a direct loading strategy, the SM–AZE–PTX/GEM at synergistic drug ratio were successfully integrated into nanovesicles without compromising the physicochemical properties of CD47p/AZE–paclitaxome-2 (Fig. 7b–f and Supplementary Tables 20 and 21). We demonstrated that CD47p/GEM/AZE–paclitaxome-2 synchronized the spatiotemporal co-delivery of GEM to tumors with significantly improved pharmacokinetics (10.4-fold longer half-life, 229.7-fold more area under the moment curve (AUMC), 10.4-fold longer mean residence time and 2.1-fold less volume of distribution (V) and 22.2-fold less clearance (CL)) (Fig. 7g–j and Supplementary Tables 22 and 23). Moreover, CD47p/GEM/AZE–paclitaxome-2 retained the ability to escape the MPS elimination well and delivered more GEM (33.7-fold) and PTX (29.6-fold) to tumors with efficient penetration and infiltration within tumors in the KPC-Luc model (Fig. 7h,j). Notably, CD47p/GEM/AZE–paclitaxome-2 not only mitigated the systemic toxicities from the combination of GEM plus Taxol (Extended Data Fig. 7), but also markedly fortified the antitumor effects in an advanced metastatic KPC-Luc PC model with notable metastasis remission compared to the co-administration of GEM and PTX formulations (Fig. 7k–p). Mechanistically, we elucidated that the improved anti-PC efficacy of co-delivery nanovesicles was attributed to the better ability to decrease the levels of cytidine deaminase (CDA), which can degrade GEM to its inactive metabolite by inducing the oxidative stress (HO-1)^35^, and the increased expression of apoptosis (cleaved caspase-3; CC-3), DNA damage (γ-H2AX) and suppressing microtubule dynamic instability (Extended Data Fig. 8)^36^.

### Co-encapsulation of CBPt for postsurgical TNBC therapy

**Fig. 8:**
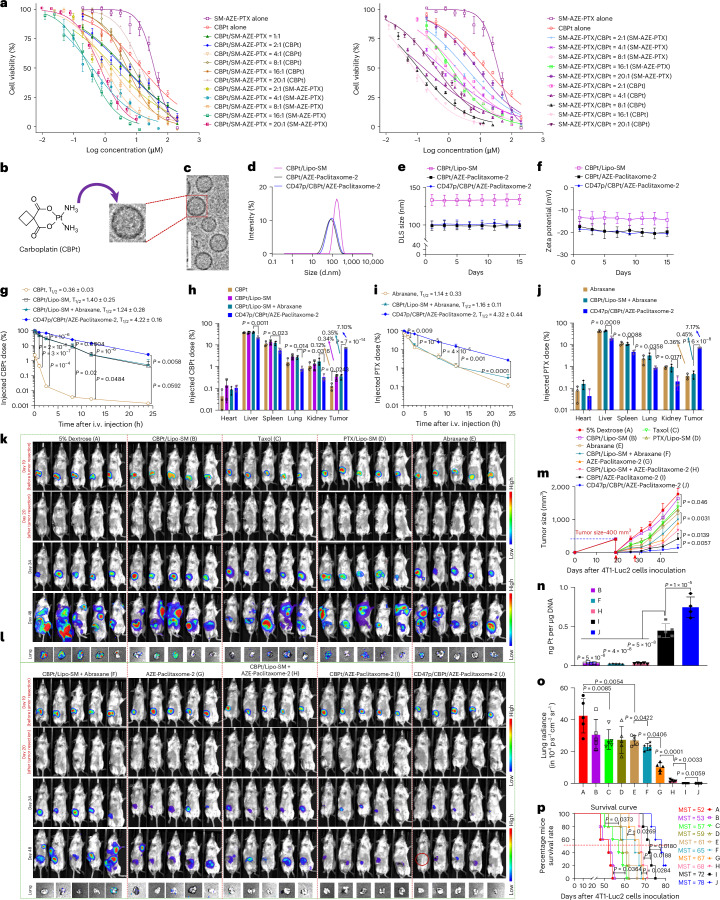
CBPt/AZE–paclitaxome-2 significantly reduced tumor growth in a postsurgical tumor relapse 4T1-Luc2 TNBC model. **a**, Drug CI of various molar ratios of CBPt and SM–AZE-PTX in 4T1-Luc2 cells by cytotoxicity test (*n* = 3 biological replicates per group). **b,c**, Schematic depicting the preparation (**b**), and cryoEM (**c**) of CD47p/CBPt/AZE–paclitaxome-2, *n* = 3 samples per group were performed independently with similar results. **d–f**, The representative distribution of DLS size via intensity (**d**), DLS size (**e**) and zeta potential (**f**) monitoring over a 15-day period at 4 °C of CBPt/Lipo–SM, CBPt/AZE–paclitaxome-2 and CD47p/CBPt/AZE–paclitaxome-2 (*n* = 3 independent experiments per group). **g**–**i**, Blood kinetics (**g**,**i**) and tissue distribution (**h**,**j**) at 24 h in orthotopic 4T1-Luc2 tumor mouse model (*n* = 3 mice per group, tumors ~400 mm^3^) after an i.v. administration of free CBPt, CBPt/Lipo–SM, CBPt/Lipo–SM + Abraxane or CD47p/CBPt/AZE–paclitaxome-2 at 7.6 mg CBPt kg^−1^ and 70 mg PTX kg^−1^ (SM–AZE–PTX/CBPt molar ratio of 4:1). **k**–**p**, 19 days after Balb/c mice were surgically inoculated with 1 × 10^5^ 4T1 cells at fourth mammary gland fat pad (*n* = 5 mice per group, tumor size ~400 mm^3^), the main tumors were removed and animal were i.v. administered with CBPt/Lipo–SM (7.6 mg kg^−1^), Taxol (20 mg PTX kg^−1^, MTD), PTX/Lipo–SM (40 mg PTX kg^−1^, MTD), Abraxane, CBPt/Lipo–SM + Abraxane, AZE–paclitaxome-2, CBPt/AZE–paclitaxome-2 or CD47p/CBPt/AZE–paclitaxome-2 at a dose of 7.6 mg CBPt kg^−1^ and 70 mg PTX kg^−1^ on day 20 and 28. **k**, Animal BLI before and after surgery. Red circle indicates tumor-free mice. **l**, Metastatic BLI of lung in mice ex vivo on day 48. **m**, Tumor growth plots, (red arrow indicates drug injection). **n**, Intratumoral platinum–DNA adducts from **m** on day 48. **o**, Quantification of BLI for lung metastasis from **l** via Lago optical imaging. **p**, Kaplan–Meier survival plots from a separate study in which mice received the same treatments as **m**. Data are presented as mean ± s.d. (**a**,**e**–**j**,**m**–**o**). One-way ANOVA with Tukey’s multiple comparisons test were used to calculate the exact *P* values and a log-rank Mantel–Cox test was utilized to compare the survival plot in the statistical analyses. Source data

**Extended Data Fig. 9:**
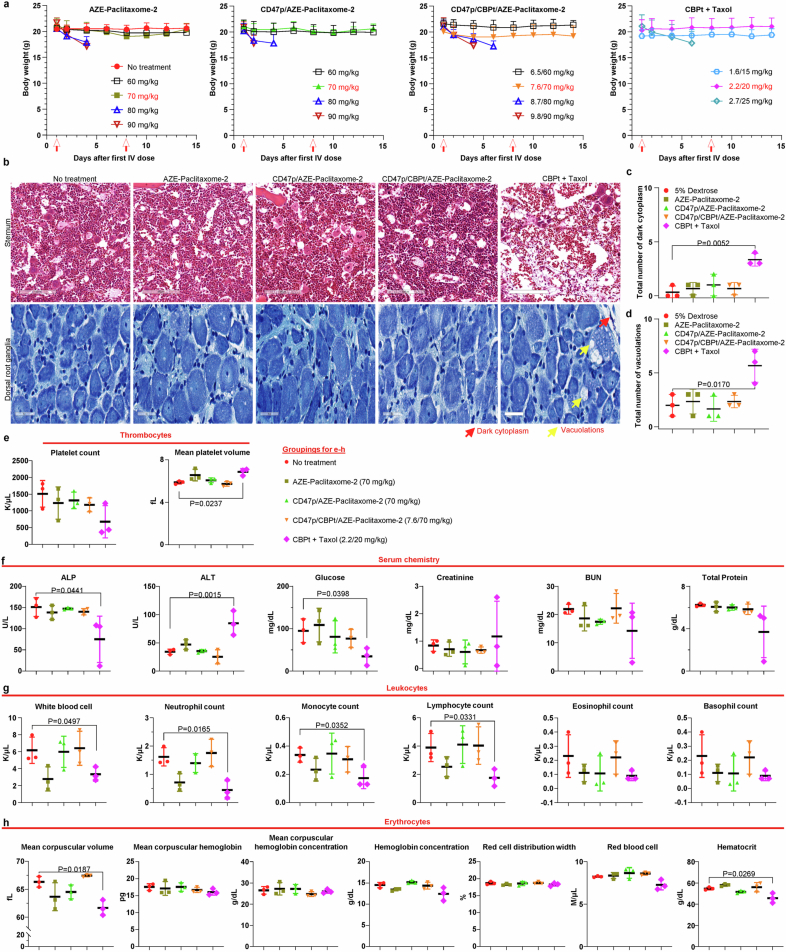
CD47p/CBPt/AZE-Paclitaxome-2 reduced systemic toxicities. **a**, The measurment of body weight in mice from the MTD evaluation of AZE-Paclitaxome-2, CD47p/AZE-Paclitaxome-2, CD47p/CBPt/AZE-Paclitaxome-2 (molar ratio SM-AZE-PTX/CBPt = 4/1) and CBPt + Taxol (molar ratio PTX/CBPt = 4/1) in healthy Balb/c mice (n = 3 mice per group) following i.v. administration via tail vein on day 1 and 8 (red arrow indicates the time of drug injection). The survival and weight of the mouse were assessed for 14 days. The MTD dose were highlighted in red color. **b-h**, representative H&E staining for sternum and toluidine blue staining for dorsal root ganglia (**b**, red arrow, dark cytoplasm; yellow arrow, vacuolations, Scale bar = 100 and 30 µm in the upper and lower panel, respectively, n = 3 mice per group), the total counting of dark cytoplasm (**c**) and vacuolations (**d**) in the whole cross-sectional area of dorsal root ganglia, thrombocytes (**e**), serum chemistry (**f**), leukocytes (**g**), erythrocytes (**h**) and examination on day 14 from MTD. Data are presented as average ± s.d. within **a** and **c-h** (n = 3 mice per group). One-way ANOVA with Tukey’s multiple comparisons test were used to calculate the exact P values in the statistical analyses. Source data

**Extended Data Fig. 10:**
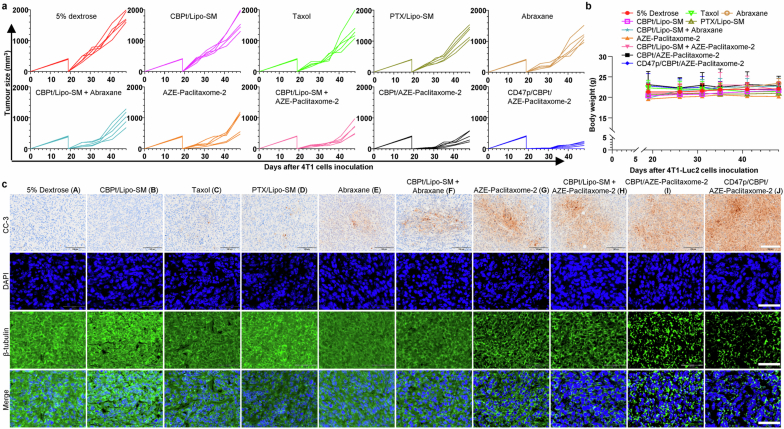
The histopathological study of postsurgical TNBC tumors after being treated with CD47p/CBPt/AZE-Paclitaxome-2. **a**,**b**, Individual tumor growth curves (**a**), body weight (**b**) from Fig. 8m (n = 5 mice per group). **c**, Representative intratumoral IHC staining for CC-3 (scale bar: 100 μm), and immunofluorescence staining for β-tubulin (scale bar = 50 μm, n = 5 tumors per group). Data are presented as average ± s.d. within **b**. Source data

Notably, in addition to GEM, PTX is combined with CBPt for the treatment of late-stage TNBC^22–24^. The PTX + CBPt combination therapy improved the efficacy, it also caused more severe systemic toxicities due to the nonspecific tissue distributions^24^. To explore whether our optimized nanovesicles can also synergize the therapeutic delivery of both CBPt and PTX and circumvent the detrimental adverse effects arising from this drug combination. We first identified the best synergistic drug molar ratio was at 4:1 for SM–AZE–PTX/CBPt (Fig. 8a and Supplementary Table 24). CBPt is hydrophilic, thus it can be directly encapsulated into the core of CD47p/AZE–paclitaxome-2, which maintained the similar size, PDI and zeta potential as the one absent CBPt (Fig. 8b–f and Supplementary Tables 25 and 26). CD47p/CBPt/AZE–paclitaxome-2 also significantly improved the systemic delivery of both CBPt and PTX in a coordinated fashion with drastically more drugs accumulated at tumors and significantly less distribution to the healthy tissues (Fig. 8g–j and Supplementary Tables 27 and 28). Notably, CD47p/GEM/AZE–paclitaxome-2 was able to significantly increase the maximal tolerated dose (MTD) for the combination of CBPt and PTX while avoiding the systemic adverse effects triggered by the co-administration of CBPt and Taxol (Extended Data Fig. 9). In a clinically relevant postsurgical 4T1-Luc2 TNBC recurrence model, CBPt/Lipo–SM failed to markedly prevent tumor relapses versus 5% dextrose. Despite the combination of CBPt/Lipo–SM and Abraxane reduced tumor growth better than monotherapy, this combination therapy was markedly outperformed by the co-delivery nanovesicles, particularly in CD47p/CBPt/AZE–paclitaxome-2 which restricted tumor growth to ~35% of the original tumor size before surgery and drastically extended mouse survival time (Fig. 8k–p and Extended Data Fig. 10a,b). In-depth mechanistic investigation deciphered that the enhanced anti-TNBC effects of CD47p/CBPt/AZE–paclitaxome-2 could be derived from the boosted platinum–DNA adducts (Fig. 8n) formation and tubulin stabilization^37^, as well as higher levels of apoptosis (CC-3) (Extended Data Fig. 10c).

## Discussion

Despite the desirable anti-neoplastic efficacy of PTX against a variety of cancers, its clinical therapeutic potential is yet to be fully unleashed due to its nonspecific toxicities, poor bioavailability and pharmacokinetics, as well as the intrinsic barriers in tumor tissues that hinder its efficient uptake and penentration^10^. A surfeit of drug delivery platform were developed with an aim to bolster the therapeutic index of PTX, but only met with very limited clinical success^3^. The only two exceptions are Taxol, a solvent-based PTX formulation, and Abraxane, a protein bound nanoparticle form of PTX. In addition to the systemic toxicities of PTX, the excipient (Cremophor EL)-induced adverse effects have plagued the use of Taxol, whereas the mediocre clinical survival benefit from Abraxane has been quite disappointing^3,11^. Liposome has contributed to most of the US FDA-approved nanotherapeutics; however, it has failed to effectively encapsulate PTX^4^. Because of hydrophobicity and its relatively large and complex molecular structure, the loading of PTX into the lipophilic bilayer can inevitably jeopardize the integrity of the liposomal membrane, resulting in poor stability and low DLC (Supplementary Tables 1 and 2), premature payload leakage and subsequent fast clearance in blood, leading to suboptimal therapeutic efficacy as seen clinically^3^. Capitalizing on the SM-derivatization strategy with PTX securely anchored in the bilayer, we proved that not only the drug loading of PTX was substantially increased, but it also endowed with much better formulation stability (Fig. 1c–e, Supplementary Tables 1–5 and Extended Data Fig. 1), preventing the unwanted drug leakage, resulting in superior pharmacokinetics and anticancer efficacy to Taxol, Abraxane and physical encapsulating of PTX liposome PTX/Lipo–SM (Figs. 2i–k and 5e–o, Extended Data Fig. 3 and Supplementary Tables 10 and 11). Because of the delayed and controlled drug release mechanism, paclitaxome also abolished the systemic side effects associated with Taxol and PTX/Lipo–SM (Fig. 2a–h). This was attributed to the slower PTX release in physiological than acidic pH, high esterase, GSH and ROS (common features of tumors; Extended Data Figs. 1s–u and 2h)^38^.

In addition, even although the EPR effect can facilitate the nanotherapeutic distribution to the periphery of tumors via leaky vasculatures^12,13^, efficient intracellular internalization with deep tissue penetration remains unattainable due to the formidable resistance induced by dense extracellular matrix and heightened interstitial fluid pressure on passive diffusion from the perivascular regions to the target cells and/or distal cells, compromising the therapeutic outcome^39^. These common phenomena present formidable barriers for nanocarrier-based therapeutic delivery for treating human diseases by hampering the concentration of the delivered drugs to a level below the therapeutic window^28^. Various strategies were developed to tackle this bottleneck like using tumor-targeting ligand and adjusting particle sizes^40–43^. Nevertheless, the clinical practice of ligand-targeted nanotherapeutics has been largely unsuccessfully, possibly because the possibility that ligand coating on nanocarrier surfaces might elicit nonspecific protein binding and untoward immunogenicity, decreasing blood circulation time and tumor uptake^44–46^. While nanocarriers with smaller sized increased the tumor penetration to some degree^43^, the efficiency was limited as they still needed to transverse the gradient of tenacious interstitial fluid pressure through a densely stuffed paracellular matrix^28^. Cationization has proven a powerful means of enabling nanoparticle penetration across multiple layers of cells for deep tumor infiltration via eliciting adsorption-mediated endocytosis and transcytosis^28,47^; however, serum components have steady nonspecific interactions with drug carriers possessing positive charge, triggering phagocytosis and resulting in rapid blood clearance^48^. The US FDA-approved liposomes have slight negative charges^49^, which are conducive to circumventing anionic serum proteins binding and avoiding opsonization to elongate blood circulation^48^. The negative charge, however, may also repel the interaction with anionic cell membranes, undermining intracellular uptake^48^. Acidic pH is a common hallmark in many tumor tissues (for example, TNBC and PC)^50^. Thus, a strategy that selectively converts anionic nanomedicine to be cationic in response to pathological acidity, while maintaining mild negative charge during circulation is of significant therapeutic impact.

To boost the tumor delivery and penetration efficiency, we anchored an ultra-pH-sensitive probe, AZE into paclitaxome. AZE has a pKa of ~6.98 and stays ‘OFF’ at physiological pH (~7.4) and can be selectively turned ‘ON’ by mild intratumoral acidic pH (~6.5)^29,50,51^. This strategy bestowed paclitaxome with an intelligent built-in charge-reversal cationization machinery that selectively triggered adsorption-mediated transcytosis by acidity-induced protonation in tumors, significantly boosting tumor delivery efficiency versus plain paclitaxome, while remaining anionic in physiological condition to avoid protein binding and opsonization^28,50,52^. Notably, the premise of this effective transcytosis was based on the caveolae/Golgi intracellular trafficking pathway rather than the traditional endolysosomes, avoiding the premature nanoparticle degradation inside cells to realize the deeper transcellular delivery within tumors (Figs. 3–5 and Extended Data Fig. 3f,g).

PEGylation has been the gold standard of enabling particulate delivery systems with ‘stealth’ effect to achieve the favorable blood circulation time; however, it has been reported that addition of PEG on nanoparticles can stimulate the complement immunity that produced anti-PEG antibodies, accelerating elimination of PEGylated nanotherapeutic following the repeated administrations, thus compromising therapeutic efficacy. The membrane protein CD47 is a reliable ‘marker of self’ that abrogates phagocytosis performed by self-macrophages through binding to receptor SIRPα^14^. To fully unlock the antitumor potential of paclitaxel, we engineered a CD47 ‘self’ peptide onto paclitaxome to substitute PEG to avoid the intrinsic drawbacks of PEG (Fig. 5a). This strategy further elongated circulation time and significantly reduced nonspecific tissue distribution versus PEG coating (in AZE–paclitaxome-2) (Fig. 5e–h and Extended Data Fig. 3f), providing a robust MPS escaping ability that complements the cationization-enabled transcytosis, and in turn bolsters therapeutic efficacy in both advanced TNBC and PC models (Figs. 5–8 and Extended Data Fig. 3j–l).

PTX’s clinical application is not limited to monotherapy. It has been established that PTX can destroy stromal density and inhibit the cytidine deaminase (CDA) that induces the GEM metabolic inactivation via upregulating oxidative stress (HO-1), thereby enhancing the therapeutic delivery of GEM into tumor sites^21,35,53,54^. Thus, PTX-based therapy has been widely combined with GEM to treat PC, especially advanced stages of disease^19^. Although GEM plus PTX combination regimen has demonstrated improved anticancer efficacy in patients with PC, the collective toxicities have hindered its clinic use^55^. Thus, it is urgently needed to establish a platform that enables the co-delivery of GEM and PTX, while mitigating the combined adverse effects. Our optimal CD47p/AZE–paclitaxome-2 can effectively encapsulate GEM at the optimal synergistic drug ratio in its interior and provide controlled and synchronized drug delivery to tumors, which boosts the antitumor efficacy by suppressing the CDA by enhancing oxidative stress, inhibiting microtubule formation, and increasing apoptosis, while minimizing the systemic side effects arising from the combination of GEM plus Taxol (Extended Data Fig. 7). Similarly, our SM–PTX nanoplatform also addressed the drawbacks (such as daunting toxicities, poor PK and tumor distribution) associated with the co-use of PTX and CBPt by co-carrying CBPt inside the nanovesicle core such that it decreased recurrence in a postsurgical TNBC model by completely eliminating metastasis (Fig. 8). Approaches that can switch ‘immune-cold’ tumors into ‘hot’ phenotype through triggering the immunogenic cancer cell death (ICD) have been validated as a robust way to potentiate the efficacy of immune checkpoint inhibitors (ICIs), which are active in a fraction of select patients with cancer^56^. Considering that PTX is a well-regarded ICD inducer^57^, in the future, the applicability of paclitaxome could be further extended to synergize with the ICIs in ‘cold’ tumors (for example, glioblastoma, ovarian, prostate and PC) that are not responsive to ICIs^58^.

In summary, we report a series of innovative and cohesive design features that are implemented into a single platform achieved by SM-derivatization nanotechnology to boost the therapeutic delivery of PTX, a promising chemotherapeutic drug that has yet to reach its maximal potential. To reiterate, we demonstrated the following findings: (1) paclitaxome-2 (particularly with AZE and CD47p) significantly improved PK and drug accumulation in tumor, and markedly enhanced antitumor activity versus Taxol and Abraxane; (2) cationization-elicited transcytosis is generalizable to various tumors as in which mild acidity is a common hallmark; (3) MPS escaping benefits endowed by CD47 ‘self’ peptide nanoengineering are better than PEGylation; (4) the ability to co-deliver and overcome the bottleneck associated with other therapeutic agents (for example, GEM and CBPt) in PTX-based combination regimens; and (5) the remarkable efficacy against advanced metastatic TNBC and PC tumors models or in clinically relevant postsurgical relapse tumor model. Through these findings, we conclude that our multipronged paclitaxome nanosystem can advance the treatment of diverse cancers with improved therapeutic outcome. Finally, the set of design strategies governing the optimal PTX delivery can also be applied to not only other small molecule drugs but also biologics (RNAs, peptide and proteins), providing a transformative toolkit to advance drug delivery and accelerate clinical translation across diverse disease in human.

## Methods

### Chemical synthesis

The detailed step-by-step approaches of chemical synthesis have been deposited in the protocols.io repository^27^.

### Ethical statement

All mice studies were approved (protocol no. 19-545) by University of Arizona (UArizona) Institutional Animal Care and Use Committee (IACUC) and followed ethical guidelines. Mice were killed as per IACUC guidelines if tumors reached maximal permitted size of 2,000 mm^3^ or if moribund (severe weight loss, weakness or inactivity). In rare cases, tumors slightly exceeded 2,000 mm^3^ on the final measurement day but were promptly addressed.

### Cells and mice

4T1 (cat. CRL-2539, UArizona Cancer Center (UACC)) and 4T1-Luc2 (cat. CRL-2539-LUC2, ATCC) cells were cultured in RPMI-1640, while KPC-Luc (cat. 153474, provided by G. Beatty, University of Pennsylvania) was maintained in DMEM. Female mice (~5 weeks old, The Jackson Laboratory) were housed in IVC systems under a 12-h light–dark cycle (7:00 on, 19:00 off), temp 68–72 °F and humidity 30–70% (as per National Institutes of Health (NIH) guidance). To ensure sex uniformity and reduce aggression-related complications, only female mice were used in this study, as male mice are more prone to fighting when housed together; however, as the analysis focuses on comparisons between treated and untreated groups, the sex of the host is considered to have minimal impact on the outcomes. Tumor size was measured using a digital caliper and calculated as (0.5 × length × width^2^).

### Preparation of paclitaxome and encapsulation of GEM or CBPt

SM, Chol, DSPE-PEG_2K_ and SM–PTX conjugate under the ratio in Supplementary Table 1 (with 0.2% w/w DSPE–Cy5) were dissolved in ethanol (5 mg PTX ml^−1^ final concentration) in a 100-ml flask. Solvent was evaporated (rotary evaporator, IKA RV 10 digital), followed by ultra-high vacuum drying (0.5 h, MaximaDry). The lipid film was hydrated with 5% dextrose (60 °C, 30 min). To load GEM or CBPt, lipid film was hydrated with 100 mg ml^−1^ gemcitabine hydrochloride or saturated carboplatin (~50 mg ml^−1^ in 5% dextrose, 60 °C, 30 min), then sonicated (ice bath, 12 min, 60 W, pulse 3/2 s on/off). Unencapsulated SM–PTX was removed by ultracentrifugation (100,000*g*, 45 min) and unencapsulated GEM or CBPt removed via PD-10 column (eluent, 5% dextrose). The nanoparticles were characterized by dynamic light scattering (DLS) (pH 6.0–7.5), CryoEM and HPLC. DLC and drug loading efficiency (DLE) were calculated using equations (1) and (2), respectively.

1 $$\frac{{\rm{weight\; of\; encapsulated\; drug}}}{{\rm{weight\; of}}\left({\rm{total\; lipids}}+{\rm{encapsulated\; drug}}\right)}\,\times \,100 \%$$

2 $$\frac{{\rm{weight\; of\; encapsulated\; drug}}}{{\rm{weight\; of\; input\; drug}}}\,\times \,100 \%$$

### The cellular uptake of various paclitaxomes

4T1-Luc2 cells (1 × 10^5^ per well) were seeded in 12-well plates to 80% confluency. For uptake studies, cells were treated with Cy5-labeled paclitaxome-2, AZE–paclitaxome-2, or AZO–paclitaxome-2 (2 μg ml^−1^ DSPE–Cy5) for 1 h at 37 °C in pH 6.5 or 7.4 medium (10% FBS). For mechanism study, endocytosis inhibitors chlorpromazine (50 μM), genistein (200 μM), cytochalasin D (5 μM), NaN₃ (10 mM) or 4 °C (energy depletion) were preincubated with the cells for 0.5 h, then treated with various Cy5-labeled formulations for 1 h. For the caveolin knockdown study, ionizable lipid (DLin-MC3-DMA), DSPC, Chol and PEG_2K_-C-DMG (49.26:10.17:39.04:1.49 molar ratio) dissolved in ethanol, various caveolin siRNA (caveolin-1, 5′-3′: GGAGAUUGACCUGGUCAACtt; antisense: GUUGACCAGGUCAAUCUCCtt; caveolin-2, 5′-3′: GGGUUUAUAAAACUGAAGUtt; antisense: ACUUCAGUUUUAUAAACCCtc; caveolin-3, 5′-3′:GGUAGAUUUUGAAGACGUGtt; antisense: CACGUCUUCAAAAUCUACCtt) target for gene caveolin-1 (siRNA ID: 60584, no. ASO2MT42, Life Technologies Corporation), caveolin-2 (siRNA ID: 72900, no. ASO2MT43, Life Technologies Corporation) and Caveolin-3 (siRNA ID: 60585, no. ASO2MT44, Life Technologies Corporation) were dissolved in citrate buffer (25 mmol l^−1^, pH 4.0). The lipid ethanol solution was rapidly mixed with the siRNA citrate buffer (1:3 v/v) under vigorous stirring at a lipid:siRNA weight ratio of 22.3:1 (N:P ≈ 6) and then dialysis against PBS (3,500 MWCO, 4 °C overnight). 4T1-Luc2 (2 × 10^5^ per well) were seeded in six-well plates, after one night, the medium was replaced with fresh medium containing 0.2 nM of siRNA and incubated at 37 °C for 24 h. After that, cells were treated with Cy5/AZE–paclitaxome-2 (DSPE–Cy5, 2 μg ml^−1^) for an additional 2 h at 37 °C. Finally, cells were washed, trypsinized and then incubated with a Zombie Violet Fixable Viability kit (BioLegend, 423114, live-cell gating), caveolin-1 Alexa Fluor 488-conjugated antibody (Bio-Techne Corporation, IC5736G, 1:10 dilution), caveolin-3 FITC-conjugated antibody (Biorbyt, orb463970, 1:100 dilution) or caveolin-2 antibody (Invitrogen, PA1-065, 1:100 dilution) for 30 min at 4 °C. After washed, goat anti-rabbit IgG (H + L) cross-adsorbed secondary antibody (Alexa Fluor 488, Invitrogen, A-11008, 1:500 dilution) was used to stain caveolin-2 for 2 h. Cells were washed and resuspended in staining buffer (eBioscience, 00-4222-26). Fluorescence was quantified via flow cytometry (BD FACSCanto II, BD FACSDiva Software, v.8.01).

### Intracellular transfer and trafficking of AZE–paclitaxome-2

The 4T1-Luc2 cells (1 × 10^6^) were plated on coverslips (i–iii) overnight. Cells in coverslip i were incubated with various Cy5/paclitaxome (DSPE–Cy5, 2 μg ml^−1^) in pH 6.5 or 7.4 medium for 4 h, washed, imaged, then co-cultured with fresh cells (coverslip ii) for 12 h and the process was repeated for transfer to coverslip iii. The cells were fixed with 4% formaldehyde (15 min, 4 °C) and the nuclei were stained with Hoechst 33342. For the trafficking study, after seeding 4T1-Luc2 cells (1 × 10^5^) on coverslips for 24 h, the cells were incubated Cy5/AZE–paclitaxome-2 (2 μg ml^−1^ DSPE–Cy5) for 6 h. Organelle staining was performed for lysosomes (LysoView 488, Fisher 70067-T), Golgi (Golgi-ID Green, Enzo, ENZ-51028-K100) and nuclei (Hoechst 33342, Life Tech 2306347) following the manufacturer’s protocol. In another assay, cells were preincubated with Exo1 (50 μM, Fisher 50-464-564) or nocodazole (10 μM, Fisher 50-196-9134) for 1 h, then added to Cy5/AZE–paclitaxome-2 (2 μg ml^−1^ DSPE–Cy5), incubated for 6 h and stained with a Golgi-ID Green assay kit (Enzo, ENZ-51028-K100). The cells were visualized using confocal microscopy (Zeiss LSM880, v.14.022.021) and colocalization was quantified (Pearson coefficient) with ImageJ (v.1.53q).

### Combination index study

KPC-Luc or 4T1-Luc2 cells (2,000 cells per well in 96-well plates) were seeded for 12 h before drug exposure. We tested combinations of SM–AZE–PTX with GEM or CBPt at various ratios: 1:1, 2:1, 4:1, 8:1, 16:1, 20:1, 1:2, 1:4, 1:8, 1:16, 1:20 (SM–AZE–PTX:GEM or CBPt) and the drugs alone were treated with the cells for 48 h. Half-maximum inhibitory concentration (IC_50_) values were measured by MTT assay. The CI was calculated using equation (3)^59^:

3 $$\frac{{{\rm{IC}}}_{50}{\rm{of\; SM}}-{\rm{AZE}}-{\rm{PTX}}}{{{\rm{IC}}}_{50}{\rm{of\; SM}}-{\rm{AZE}}-{\rm{PTX\; alone}}}+\,\frac{{{\rm{IC}}}_{50}{\rm{of\; GEM\; or\; CBPt}}}{{{\rm{IC}}}_{50}{\rm{of\; GEM\; or\; CBPt\; alone}}}$$

CI < 1, synergy; CI = 1, additive; CI > 1, antagonism.

### Pharmacokinetics and biodistribution

Taxol (20 mg PTX kg^−1^), Abraxane (70 mg PTX kg^−1^), PTX/Lipo–SM (40 mg PTX kg^−1^), paclitaxome-1 (70 mg PTX kg^−1^), paclitaxome-2 (70 mg PTX kg^−1^), paclitaxome-3 (70 mg PTX kg^−1^), GEM (12.3 mg kg^−1^), GEM/Lipo–SM (12.3 mg kg^−1^), GEM/Lipo–SM (12.3 mg kg^−1^) + Abraxane (70 mg PTX kg^−1^), CD47p/GEM/AZE–paclitaxome-2 (12.3 mg GEM kg^−1^, 70 mg PTX kg^−1^), CBPt (7.6 mg kg^−1^), CBPt/Lipo–SM (7.6 mg kg^−1^), CBPt/Lipo–SM (7.6 mg kg^−1^) + Abraxane (70 mg PTX kg^−1^) or CD47p/CBPt/AZE–paclitaxome-2 (7.6 mg CBPt kg^−1^, 70 mg PTX kg^−1^) were i.v. injected to orthotopic 4T1-Luc2 tumor-bearing mice (*n* = 3, tumor size ~300 mm^3^) or orthotopic KPC-Luc tumor-bearing mice (*n* = 3, tumor weight ~400 mg), respectively. Blood sampling was carried out at 0.083, 0.33, 1, 2.5, 8, 12 and 24 h post-dose in BD Microtainer tubes and then digested in methanol. Tumor and organs were collected and homogenized in acidified methanol (0.075 M HCl, 900 μl per 100 mg tissue). The samples were analyzed by HPLC or ICP-MS and PKSolver v.2.0 was used for parameter calculations^60^.

### Visualization of biodistribution and deep tumor penetration

Cy5-labeled paclitaxome (70 mg PTX kg^−1^) or Cy5/PTX/Lipo–SM (40 mg PTX kg^−1^) were intravenously injected into orthotopic 4T1-Luc2 or KPC-Luc tumors mice (*n* = 3 mice/group, ~400 mg). At 24 h later, the tumor and organs were collected and imaged under Lago optical imager (Ex640/Em690 nm; Aura software v.3.2.0). Tumors were snap-frozen in acetone/dry ice mixture and sectioned (5 µm) for blood vessels (anti-CD31 (Abcam, ab28364, 1:50) + AF488 secondary (Abcam, ab150073, 1:400 dilution)) and cellular nuclei (DAPI) staining, and then visualized by confocal microscopy (Zeiss LSM880, v.14.022.021).

### Differential scanning calorimetry

Various paclitaxomes (2.0 mg lipid ml^−1^, in 400 µl degassing deionized water (DI) water) were analyzed using MicroCal VP-capillary DSC (10–60 °C, 1 °C min^−1^) to measure the DSC data based on our previously established method^61^. Data were converted to molar heat capacity using VPViewer 2000 and MicroCal software (LLC Cap DSC, v.Origin70-L3).

### Molecular dynamic simulation

Molar ratio of 70.19% SM, 21.73% SM–CSS-PTX, 3% Chol and 5.08% DSPE-PEG_2K_ bilayer simulation were performed in GROMACS (CHARMM36 forcefield, TIP3P water, v.2019.3)^62,63^ under the conditions of 298.15 K, 1 bar (Parrinello–Rahman barostat) and 0.002 ps time step^64,65^. All components were independently coupled to a V-rescale thermostat (*τ* = 20,000 ps). Long-range electrostatics were solved with Particle Mesh Ewald under periodic boundary conditions. Bilayer stability was evaluated via the thickness of the membrane, average area of the lipid and root mean square deviation. Order parameter *S*z and order parameter *S* were determined using equation (4).

4 $$\frac{1}{2}\left(3{\cos }^{2}\theta -1\right)$$

*S* (θ = C − H/bilayer-normal angle) reflects lipid alignment, with higher values indicating greater order.

### Cryogenic transmission electron microscopy

Liposomes (3 µl) on C-Flat grids (Protochips) were Vitrobot-blotted (100% RH, 3–6 s) and cryo-immobilized in ethane. Samples were examined by cryo-TEM (Philips TF20, 120 keV) using a Gatan CT3500 cryoholder maintained at −180 °C. Data were collected using a TVIPS XF416 CMOS camera and processed with manufacturer-provided EMMenu software (TVIPS, v.3.1.5).

### Drug release kinetics of paclitaxomes

The release of various paclitaxomes were measured via dialysis (PBS/0.5% Tween 80) at different pH (5-7.4), with esterase (10 U), GSH (10 mM) or H_2_O_2_ (10 mM). The 5 mg PTX ml^−1^ paclitaxome solutions (4 ml) were dialyzed (12 kDa) against 200 ml release medium at 37 °C with stirring at 100 rpm. At the designated time points, 5 μl (retentate) or 100 μl (dialysate) samples were analyzed by HPLC (227 nm) to quantify SM–PTX and PTX.

### Penetration of paclitaxome-2 in multicellular spheroids

Single-cell suspensions of 4T1-Luc2 (5,000 cells/well) in Collagen I (5 μg ml^−1^, Thermo, A10483-01) were centrifuged (450*g*, 10 min, 4 °C) in U-bottom 96-well plates (Thermo, 174925) to initiate spheroid formation. After 96 h maturation, spheroids were dosed with Cy5-labeled formulations (2 μg ml^−1^) in pH 6.5 or 7.4 medium. At designated intervals, spheroids were PBS-washed and visualized under confocal microscopy (Zeiss LSM880, v.14.022.021).

### Maximum tolerated dose

Formulations were administered intravenously to Balb/c or B6129SF1/J mice (*n* = 3 mice/group) across escalating doses as indicated. Mice were monitored for 14 days to determine MTD, defined as the dose inducing neither mortality nor >15% body weight loss, with maintained normal appearance status. At the end point (day 14), serum obtained by cardiac puncture (lithium heparin tubes) was centrifuged (2,000*g*, 10 min, 4 °C) and analyzed for clinical chemistry (Liasys 330, UArizona Animal Care Pathology Core). Complete blood counts (Hemavet 950FS) were performed on K_2_EDTA-anticoagulated whole blood. Sternum and dorsal root ganglia were fixed for histopathological assessment by UACC’s Tissue Acquisition and Cellular/Molecular Analysis Shared Resource core. Dorsal root ganglia cross-sections were digitally scanned (Leica Aperio Versa 200), with cytoplasmic abnormalities and vacuolations quantified using Aperio ImageScope software (v.12.4.3).

### Measure the concentration of platinum by ICP-MS

Carboplatin-derived platinum was quantified using ICP-MS (Agilent 8900 with MassHunter v.5.1), following quality assurance/quality control protocols adapted from US EPA Method 200.8. Calibration standards were prepared from certified Pt stock solutions (Inorganic Ventures) in 2% ultrapure HCl (VWR AriStar Plus), with seven-point curves (*R*^2^ > 0.995). Biological samples were microwave-digested before analysis, and then diluted 100-fold in 2% HCl, analyzed with continuing calibration verification verification after calibration and quality control sample (QCS) checks every 12 samples (per EPA 200.8). QCS solutions (NIST SRM 1643e or High Purity Standards) were analyzed with 90–110% recovery criteria. An internal standard (Rh or In) was introduced online via mixing tee for signal normalization.

### The measurement of pKa for SM–AZO–PTX and SM–AZE–PTX

The pKa of SM–AZO–PTX and SM–AZE–PTX was determined as previously described^51,66^. In brief, conjugates (20 mg) were dissolved in 0.04 M HCl/150 mM NaCl (sonication), then diluted to 2 mg ml^−1^ with 150 mM NaCl for analysis. The solution was titrated from pH 2 to 11 by stepwise addition of 0.02 M NaOH (5–10-µl increments) while stirring, with pH recorded using a calibrated Mettler Toledo microelectrode. Protonation at 0% and 100% was set as the first-derivative extremes of the pH titration curves.

### Immunofluorescence staining and immunohistochemistry

Tumor samples were fixed in 4% paraformaldehyde (overnight), paraffin-embedded and cut into 4-μm sections. Tumor sections were immunostained with β-tubulin Alexa Fluor 488-conjugated antibody (1:100 dilution, Cell Signaling, 3623S) and DAPI, then imaged using an Olympus VS200 slide scanner and processed with OlyVIA software (v.4.1). For the IHC study, after deparaffinization (60 °C) and antigen retrieval (1 mM citrate, pH 6, 98 °C), slides were treated with 3% H_2_O_2_ (5 min) and incubated with primary antibodies (15–50 min): cleaved caspase-3 (1:300 dilution, Cell Signaling, 9664S), γ-H2AX (1:400 dilution, Abcam ab22551), HO-1 (1:400 dilution, Abcam ab52947) and CDA (1:300 dilution, Invitrogen PA5-95616) on a Leica Bond RXm autostainer. After secondary antibody (HRP polymer), slides were developed with DAB (10 min), counterstained with hematoxylin (5 min), dehydrated (ethanol/xylene) and coverslipped using Bond Refine Detection kit reagents (Leica, DS9800). Digital whole-slide images were acquired using an Olympus VS200 scanner and analyzed with OlyVIA software (v.4.1).

### Intratumoral platinum–DNA adduct measurement

Pt–DNA adducts were quantified as described^67^. Tumor DNA was extracted (proteinase K/phenol–chloroform) and dissolved in 10 mM Tris-HCl (pH 7.4) with 1 mM EDTA. DNA purity was confirmed by UV spectroscopy (A260/A280 > 1.8) with A320 background subtraction. Samples were RNase A-treated (1 µg ml^−1^, 2 h), re-extracted and DNA concentrations were calculated as (A260–A320) × 50 µg ml^−1^. Platinum levels were determined by ICP-MS.

### Orthotopic metastatic KPC-Luc PC model

B6129SF1/J mice were anesthetized (isoflurane), administered buprenorphine SR (1.0 mg kg^−1^, subcutaneously (s.c.)) and shaved abdominally before orthotopic KPC-Luc tumor implantation. After sterilizing the abdomen (ethanol/betadine), a 0.5–0.7 cm incision exposed the pancreas. KPC-Luc cells (2 × 10^6^ in 50 μl RPMI:Matrigel 3:1) were injected into the pancreatic tail vein using a 26G needle. The injection site was sterilized with 70% ethanol to eliminate potential tumor cell leakage. After repositioning the pancreas, fascial and skin layers were sutured (6-0 PDS II and 5-0 PROLENE, respectively). Mice recovered on heating pads with monitoring until ambulatory. Orthotopic KPC-Luc mice (*n* = 5 mice per group, ~400 mg tumors) were randomized into indicated treatment groups at day 10 or 11 post-inoculation. Mice received the following i.v. treatment: 5% dextrose, GEM/Lipo–SM (12.3 mg kg^−1^), Abraxane (70 mg kg^−1^), GEM/Lipo–SM (12.3 mg kg^−1^) combined with Abraxane (70 mg kg^−1^), CD47p/AZE–paclitaxome-2 (70 mg PTX kg^−1^), GEM/Lipo–SM (12.3 mg kg^−1^) combined with CD47p/AZE–paclitaxome-2 (70 mg PTX kg^−1^) or CD47p/GEM/AZE–paclitaxome-2 (12.3 mg GEM kg^−1^, 70 mg PTX kg^−1^) on day 11 and 19 in Fig. 7k–p; 5% dextrose, Taxol (20 mg kg^−1^), Abraxane (70 mg kg^−1^), PTX/Lipo–SM (40 mg PTX kg^−1^), paclitaxome-2 (70 mg PTX kg^−1^), AZO–paclitaxome-2 (70 mg PTX kg^−1^), AZE–paclitaxome-2 (70 mg PTX kg^−1^) and CD47p/paclitaxome-2 (70 mg PTX kg^−1^) in Extended Data Fig. 3j–l; 5% dextrose, SM–AZE/paclitaxome-2 (70 mg PTX kg^−1^), paclitaxome-2 (70 mg PTX kg^−1^), AZE–paclitaxome-2 (70 mg PTX kg^−1^) and CD47p/AZE–paclitaxome-2 (70 mg PTX kg^−1^) on day 10 and 18 in Extended Data Fig. 5; 5% dextrose, CP–PTX (70 mg PTX kg^−1^), PGG–PTX (70 mg PTX kg^−1^), paclitaxome-2 (70 mg PTX kg^−1^), AZE–paclitaxome-2 (70 mg PTX kg^−1^) and CD47p/AZE–paclitaxome-2 (70 mg PTX kg^−1^) on day 10 and 18 in Extended Data Fig. 6. Whole-body tumor burden was assessed by bioluminescence imaging (Lago system, Aura v.3.2.0) following intraperitoneal (i.p.) injection of 150 mg kg^−1^ d-luciferin (GoldBio). Radiance values (p s^−1^ cm^−2^ sr^−1^) were quantified at the indicated time points, with parallel monitoring of body weight and survival. For metastasis quantification, organs were dissected immediately after d-luciferin injection and imaged ex vivo using the Lago system.

### Orthotopic 4T1, 4T1-Luc2 and surgical 4T1-Luc2 TNBC

Orthotopic 4T1 or 4T1-Luc2 tumors were established in Balb/c mice (*n* = 5 mice per group) under aseptic conditions following buprenorphine SR premedication (1.0 mg kg^−1^ s.c.) and isoflurane anesthesia. The surgical site was sterilized with three alternating betadine and 70% ethanol scrubs, a 0.5-cm abdominal incision exposed the fourth mammary fat pad, where 50 μl of RPMI/Matrigel (3:1) containing 1 × 10^5^ 4T1 or 2 × 10^5^ 4T1-Luc2 cells were injected using a 26G needle. After repositioning and ethanol sterilization, the skin was closed with wound clips (BD). Mice recovered on heating pads with monitoring until ambulatory. In Fig. 2i–k, mice (4T1 tumors size ~100 mm^3^) received i.v. administered 5% dextrose, Taxol (20 mg kg^−1^), Abraxane (20 mg kg^−1^), PTX/Lipo–SM (20 mg PTX kg^−1^) and various paclitaxome (20 mg PTX kg^−1^) on day 9 and 19. In Fig. 5j–o, the mice (4T1-Luc2 tumor size ~300 mm^3^) received i.v. administered 5% dextrose, Taxol (20 mg kg^−1^), Abraxane (70 mg kg^−1^), PTX/Lipo–SM (40 mg PTX kg^−1^), paclitaxome-2 (70 mg PTX kg^−1^), AZO–paclitaxome-2 (70 mg PTX kg^−1^), AZE–paclitaxome-2 (70 mg PTX kg^−1^) and CD47p/paclitaxome-2 (70 mg PTX kg^−1^), on day 16 and 24. In Fig. 8, On day 19 post-inoculation (tumors ~400 mm³), primary tumors were surgically resected under anesthesia with intentional retention of ~1% residual tissue. Wounds were closed with autoclips (BD), and mice received 5% dextrose, CBPt/Lipo–SM (7.6 mg kg^−1^), Taxol (20 mg PTX kg^−1^), PTX/Lipo–SM (40 mg PTX kg^−1^), Abraxane (70 mg PTX kg^−1^), CBPt/Lipo–SM (7.6 mg kg^−1^) combined with Abraxane (70 mg PTX kg^−1^), AZE–paclitaxome-2 (70 mg PTX kg^−1^), CBPt/Lipo–SM (7.6 mg kg^−1^) combined with AZE–paclitaxome-2 (70 mg PTX kg^−1^), CBPt/AZE–paclitaxome-2 (7.6 mg CBPt kg^−1^, 70 mg PTX kg^−1^), CD47p/CBPt/AZE–paclitaxome-2 (7.6 mg CBPt kg^−1^, 70 mg PTX kg^−1^) on day 20 and 28. Tumor size (by caliper) and whole-body burden (by Lago imaging after 150 mg kg^−1^ d-luciferin i.p.) were tracked. Lung metastases were analyzed ex vivo. Survival was monitored in a parallel study.

### Statistics and reproducibility

The data in graphs are represented as mean ± s.d. Exact *P* values in statistical analyses were determined by unpaired two-tailed *t*-tests (two groups), one-way analysis of variance with Tukey’s multiple comparisons (three or more groups) or log-rank Mantel–Cox test (survival) using GraphPad Prism v.8.0. Statistical significance was set at *P* < 0.05. No statistical method was used to predetermine sample sizes but our sample sizes were similar to those reported in previous publications^26^. Data distribution was assumed to be normal, but this was not formally tested. Individual data points are represented as dots in graphs and no data or animals were excluded. Before treatment, animals with similar tumor sizes and body weights were randomized among littermates. The serum chemistry, hematological counts, CryoEM and ICP-MS were conducted by independent scientists, who were unaware of the treatment conditions, in respective core facilities. For other assays, the investigators were not blinded to allocation during experimentation and outcome assessment.

### Reporting summary

Further information on research design is available in the Nature Portfolio Reporting Summary linked to this article.

## Supplementary information

Supplementary Information Supplementary Figs. 1 and 2.

Reporting Summary

Supplementary Tables Supplementary Tables 1–28.

## Source data

Source Data Fig. 1–8 and Source Data Extended Data Fig. 1–10 Statistical source data.

## Data Availability

Source data for all figures and extended data figures have been provided in Source Data files. All other data supporting the findings of this study are available within the article, Supplementary Information and from the corresponding author on reasonable request. Source data are provided with this paper.
