# Supplementary material for: A sphingolipid-derived paclitaxel nanovesicle enhances efficacy of combination therapies in triple-negative breast cancer and pancreatic cancer
Source: Nat Cancer. 2025 Aug 21;6(10):1734–53. doi: 10.1038/s43018-025-01029-7 (PMC12559008; doi:10.1038/s43018-025-01029-7)
Supplement: Supplementary file 1 — Supplementary Figs. 1 and 2. [file 43018_2025_1029_MOESM1_ESM.pdf]

nature cancer

Supplementary information

https://doi.org/10.1038/s43018-025-01029-7

A sphingolipid-derived paclitaxel nanovesicle enhances efficacy of combination therapies in triple-negative breast cancer and pancreatic cancer

In the format provided by the authors and unedited

Table of Contents:

Supplementary Figure 1…………………………………………….……………………...2

Supplementary Figure 2……………………………………….……….…………………..3

The ^{1}H NMR spectra of various Paclitaxomes and its compositions

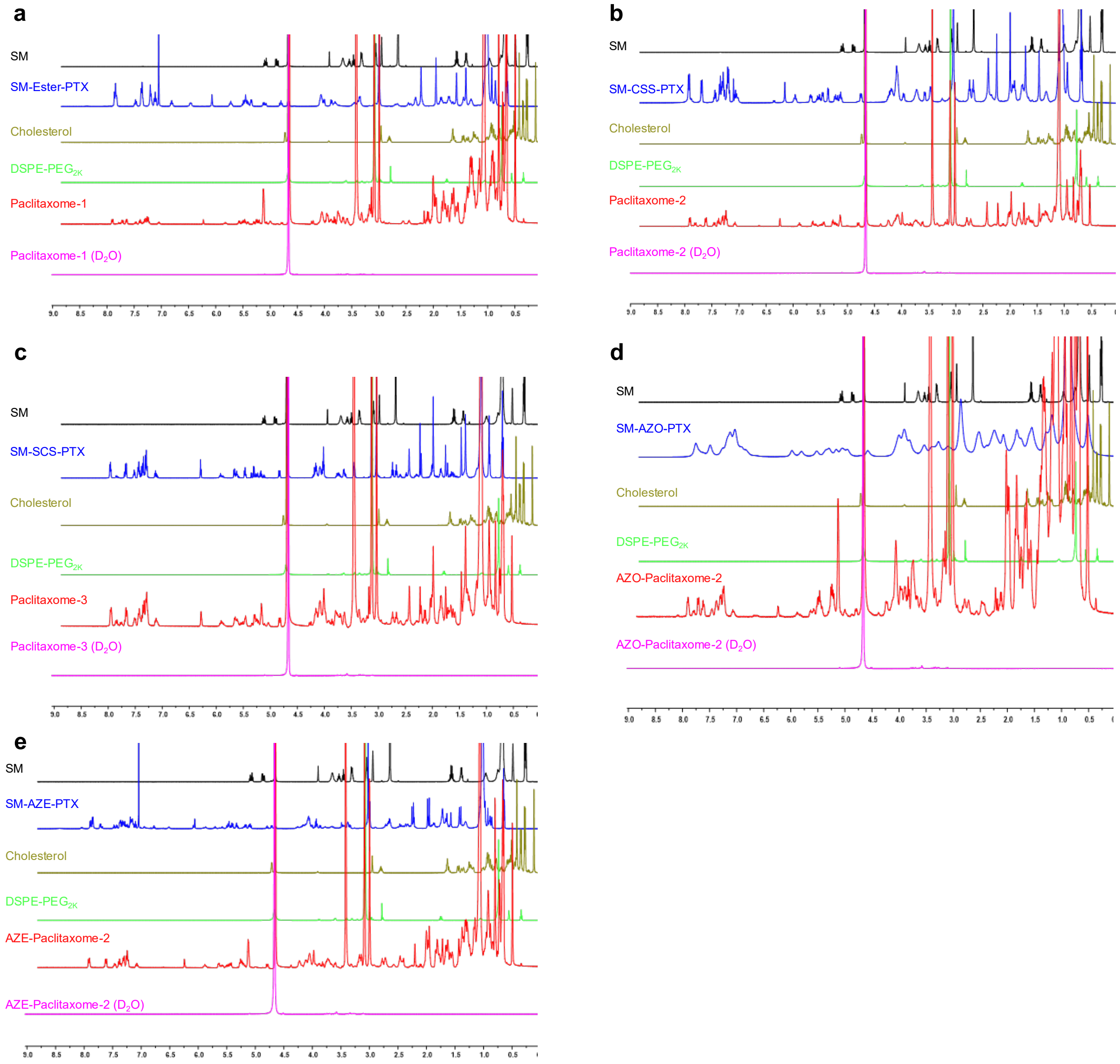

Supplementary Fig. 1. **a**, ^{1}H NMR spectra of Paclitaxome-1 (**a**), Paclitaxome-2 (**b**), Paclitaxome-3 (**c**), AZO-Paclitaxome-2 (**d**) and AZE-Paclitaxome-2 (**e**) in D_2O, and their respective SM-PTX conjugate, SM, Cholesterol, and DSPE-PEG$_{2K}$ in MeOD. The typical proton spectra were shown for SM-PTX, SM, Cholesterol, DSPE-PEG$_{2K}$ in MeOD due to their free dispersion in this solvent. Paclitaxome in MeOD expressed all typical proton signals for each individual constituent. However, when collected in D_2O, nearly all the proton signals from individual components in Paclitaxome were all suppressed, which can be attributed to their spontaneous self-assembly into Paclitaxome in **Fig. 1** and **Fig. 3**.

The gating methods of flow cytometry

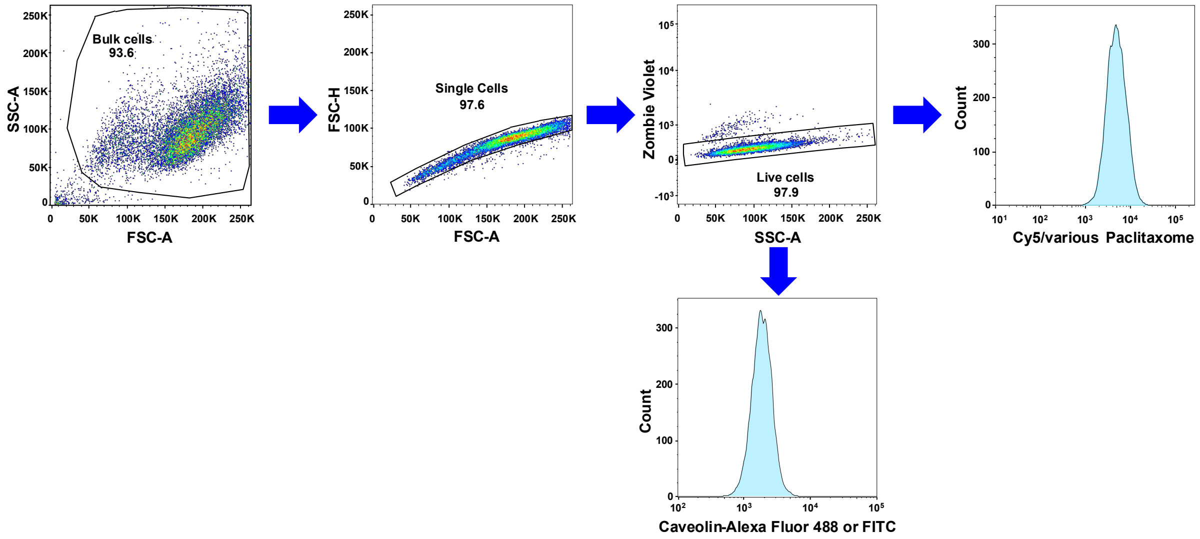

Supplementary Fig. 2. Gating methods used to define cellular uptake of Cy5 labeled various Paclitaxomes and the expression of various Caveolin in **Fig. 4**.
