# Supplementary material for: A sphingolipid-derived paclitaxel nanovesicle enhances efficacy of combination therapies in triple-negative breast cancer and pancreatic cancer
Source: Nat Cancer. 2025 Aug 21;6(10):1734–53. doi: 10.1038/s43018-025-01029-7 (PMC12559008; doi:10.1038/s43018-025-01029-7)
Supplement: Supplementary file 2 — Reporting Summary [file 43018_2025_1029_MOESM2_ESM.pdf]

nature portfolio

Corresponding author(s): Jianqin Lu

Last updated by author(s): Jun 13, 2025

Reporting Summary

Nature Portfolio wishes to improve the reproducibility of the work that we publish. This form provides structure for consistency and transparency in reporting. For further information on Nature Portfolio policies, see our Editorial Policies and the Editorial Policy Checklist.

Statistics

For all statistical analyses, confirm that the following items are present in the figure legend, table legend, main text, or Methods section.

n/a	Confirmed	
☐	☒	The exact sample size (*n*) for each experimental group/condition, given as a discrete number and unit of measurement
☐	☒	A statement on whether measurements were taken from distinct samples or whether the same sample was measured repeatedly
☐	☒	The statistical test(s) used AND whether they are one- or two-sided<br>*Only common tests should be described solely by name; describe more complex techniques in the Methods section.*
☐	☒	A description of all covariates tested
☐	☒	A description of any assumptions or corrections, such as tests of normality and adjustment for multiple comparisons
☐	☒	A full description of the statistical parameters including central tendency (e.g. means) or other basic estimates (e.g. regression coefficient) AND variation (e.g. standard deviation) or associated estimates of uncertainty (e.g. confidence intervals)
☐	☒	For null hypothesis testing, the test statistic (e.g. *F*, *t*, *r*) with confidence intervals, effect sizes, degrees of freedom and *P* value noted<br>*Give P values as exact values whenever suitable.*
☐	☒	For Bayesian analysis, information on the choice of priors and Markov chain Monte Carlo settings
☐	☒	For hierarchical and complex designs, identification of the appropriate level for tests and full reporting of outcomes
☐	☒	Estimates of effect sizes (e.g. Cohen's *d*, Pearson's *r*), indicating how they were calculated

Our web collection on statistics for biologists contains articles on many of the points above.

Software and code

Policy information about availability of computer code

Data collection	NMR spectra were acquired by Bruker topspin software (v. 2.1). HPLC spectra were acquired by ChemStation Rev.A. software (v. 10.01, Agilent Technology). DLS size and zeta potential data were acquired by Zetasizer software (v. 7.13). Cryo-EM image were acquired by Tecnai User Interface software (v. 3.1.5) and EMMenu. Histology images were acquired by Olympus VS200 slide scanner (version OlyVIA V4.1). H&E imaging were acquired by Leica DMI6000B microscope with a Leica DFC450 color camera and the Leica LAS X 3.7 software. Ultraviolet absorption and fluorescence were acquired by SoftMax® Pro (v. 7.1.0). Serum chemistry and hematological counts were acquired by Liasys 330 and Hemavet 950FS, respectively. Immunofluorescence images were acquired by Zeiss LSM880 inverted confocal microscope (Zen Black software (v. 14.022.021)). Pharmacokinetic parameters were acquired by PKSolver software (version 2.0). In vivo bioluminescence and fluorescence images were acquired by Aura 64 Bit Analysis software (v. 3.2.0). Histology images were acquired by Olympus VS200 slide scanner (version OlyVIA V4.1). H&E imaging were acquired by Leica DMI6000B microscope with a Leica DFC450 color camera and the Leica LAS X 3.7 software. Molecular dynamic simulation were performed on GROMACS software (v. 2019.3). DSC data were acquired by VPViewer 2000 (v. 2.65). Flow cytometry data were acquired by BD FACSCanto II (BD FACSDiva Software, version 8.01). Histology images were acquired by Aperio ImageScope software (version 12.4.3.5008).
Data analysis	NMR data spectra were analyzed by MestReNova (v. 6.0.2). HPLC data and spectra were analyzed by ChemStation Rev.A. software (v. 10.01, Agilent Technology). In vivo bioluminescence and fluorescence images were analysed by Aura 64 Bit Analysis software (v. 3.2.0). All statistical analyses were performed with Graphpad Prism 8. Fluorescence intensity was quantified by ImageJ software (Version.1.53q). Flow cytometry were analyzed by FlowJo software (version 10.0.7, TreeStar)

For manuscripts utilizing custom algorithms or software that are central to the research but not yet described in published literature, software must be made available to editors and reviewers. We strongly encourage code deposition in a community repository (e.g. GitHub). See the Nature Portfolio guidelines for submitting code & software for further information.

Data

Policy information about availability of data

All manuscripts must include a data availability statement. This statement should provide the following information, where applicable:

- Accession codes, unique identifiers, or web links for publicly available datasets
- A description of any restrictions on data availability
- For clinical datasets or third party data, please ensure that the statement adheres to our policy

Source data for all Figures and Extended Data Figures have been provided in Source Data files. All other data supporting the findings of this study are available within the article, Supplementary Information and from the corresponding author on reasonable request.

Research involving human participants, their data, or biological material

Policy information about studies with human participants or human data. See also policy information about sex, gender (identity/presentation), and sexual orientation and race, ethnicity and racism.

Reporting on sex and gender	NA
Reporting on race, ethnicity, or other socially relevant groupings	NA
Population characteristics	NA
Recruitment	NA
Ethics oversight	NA

Note that full information on the approval of the study protocol must also be provided in the manuscript.

Field-specific reporting

Please select the one below that is the best fit for your research. If you are not sure, read the appropriate sections before making your selection.

☒ Life sciences ☐ Behavioural & social sciences ☐ Ecological, evolutionary & environmental sciences

For a reference copy of the document with all sections, see nature.com/documents/nr-reporting-summary-flat.pdf

Life sciences study design

All studies must disclose on these points even when the disclosure is negative.

Sample size	No statistical method was used to predetermine sample sizes but our sample sizes are similar to those reported in previous publications (Nat. Nanotechnol. 2021,16(10):1130-1140)
Data exclusions	No data or animals were excluded.
Replication	Most in vitro experiments were have 3 biologically independent samples or repeated independently for at least 3 times. All in vivo studies were repeated at least 5 mice per group. The detailed information is also described in the figure legends and methods section.
Randomization	Prior to treatment, animals with similar tumor sizes and body weights were randomized among littermates.
Blinding	The serum chemistry, hematological counts, Cryo-EM and ICP-MS were conducted by independent scientists, who were unaware of the treatment conditions, in respective core facilities. For other assays, the investigators were not blinded to allocation during experimentation and outcome assessment.

Reporting for specific materials, systems and methods

We require information from authors about some types of materials, experimental systems and methods used in many studies. Here, indicate whether each material, system or method listed is relevant to your study. If you are not sure if a list item applies to your research, read the appropriate section before selecting a response.

Materials & experimental systems

n/a	Involved in the study
☐	☒ Antibodies
☐	☒ Eukaryotic cell lines
☒	☐ Palaeontology and archaeology
☐	☒ Animals and other organisms
☒	☐ Clinical data
☒	☐ Dual use research of concern
☒	☐ Plants

Methods

n/a	Involved in the study
☒	☐ ChIP-seq
☐	☒ Flow cytometry
☒	☐ MRI-based neuroimaging

Antibodies

Antibodies used

Immunofluorescence Experiments:
Anti-CD31 (a.k.a. PECAM-1) antibody (Abcam, Cat#: ab28364, Rabbit polyclonal to CD31, dilution: 1/50)
Alexa Fluor 488-conjugated secondary antibody (Abcam, Cat#: ab150073, Donkey polyclonal Secondary Antibody to Rabbit IgG - H&L dilution: 1/400)
GOLGI ID® Green assay kit (Enzo Life Sciences, Inc, Cat#: ENZ-51028-K100, dilution: 1/50),
LysoView 488 (Biotium, Cat#: 70067-T, dilution: 1/1000)
β-Tubulin Rabbit mAb (Cell Signaling, Cat#: 3623S, 9F3, Alexa Fluor® 488 Conjugate, 1/100)

Immunohistochemistry Experiments:
anti cleaved caspase-3 (Cell Signaling, 5A1E, Cat#: 9664S, dilution: 1/300)
anti-gamma H2A.X (Abcam, 3F2, Cat#: ab22551, dilution: 1/400)
anti-Heme Oxygenase 1 (Abcam, EP1391Y, Cat#: ab52947, dilution: 1/400)
anti-CDA (Invitrogen, Cat#: PA5-95616, dilution: 1/300)

Flow cytometry:
Caveolin-1 Alexa Fluor® 488-conjugated antibody (Bio-Techne Corporation, #IC5736G, 1:10 dilution)
Caveolin 2 antibody (Invitrogen, #PA1-065, 1:100 dilution, 1:100 dilution)
Caveolin-3 FITC-conjugated antibody (Biorbyt, #orb463970, 1:100 dilution)
Alexa Fluor™ 488-conjugated goat anti-rabbit IgG (H+L) cross-adsorbed secondary antibody (Invitrogen, # A-11008, 1:500 dilution)

Validation

All antibodies were verified by the supplier's websites and/or results presented in the manuscript.
The detailed information are listed as below:
Immunofluorescence Experiments:
1) Anti-CD31; rabbit; suitable for IHC-P; reacts with human, mouse, pig
2) β-Tubulin Rabbit mAb; Rabbit; suitable for IF; reacts with Human, Mouse, Rat, Monkey, Zebrafish, Bovine.

Immunohistochemistry Experiments:
1) anti cleaved caspase-3, rabbit; suitable for IHC-P; reacts with Mouse, Rat, Monkey.
2) anti-gamma H2A.X, mouse; suitable for IHC-P; reacts with Mouse, human.
3) anti-Heme Oxygenase 1, rabbit; suitable for IHC-P; reacts with Mouse, human.
4) anti-CDA, rabbit; suitable for IHC-P; reacts with Mouse, human.

Flow cytometry:
1) Caveolin-1 Alexa Fluor® 488-conjugated antibody; suitable for Flow cytometry; reacts with Mouse, Rat, human.
2) Caveolin 2 antibody; suitable for Flow cytometry; suitable for IF, WB; reacts with Mouse, Rat.
3) Caveolin-3 FITC-conjugated antibody; suitable for IF, Flow cytometry; reacts with Mouse, Rat.
4) Alexa Fluor™ 488-conjugated goat anti-rabbit IgG (H+L) cross-adsorbed secondary antibody; Goat / IgG; suitable for Flow cytometry; against human IgG, human serum, mouse IgG, mouse serum and bovine serum

Eukaryotic cell lines

Policy information about cell lines and Sex and Gender in Research

Cell line source(s)

Mouse breast cancer cells 4T1 (Cat. CRL-2539™, female) were obtained from UArizona Cancer Center. Mouse breast cancer cells 4T1-Luc2 (Cat. CRL-2539-LUC2, female) was obtained from American Type Culture Collection (ATCC). Mouse pancreatic cancer cells KPC-Luc (Cat. 153474) was provided by Professor Gregory Beatty at University of Pennsylvania, who's lab genetically engineered the cell lines.

Authentication

Cell lines were used without any modification once received from respective suppliers and therefore were not authenticated.

Mycoplasma contamination

All cell lines were regularly tested for mycoplasma contamination and no mycoplasma contamination was observed.

Commonly misidentified lines (See ICLAC register)	None of the cell lines used are listed in the ICLAC list.

Animals and other research organisms

Policy information about studies involving animals; ARRIVE guidelines recommended for reporting animal research, and Sex and Gender in Research

Laboratory animals	5 weeks old, female Balb/c and B6129J1 mice were purchased from Jackson laboratory. Mice were housed in Standard Individually Ventilated Caging (IVC). The Light cycle is 12/12 – 12 hours light/12 hours dark with 7am on-7pm off. The Temperature is maintained between 68°-72°F and the humidity is between 30-70% per the NIH Guide.
Wild animals	The study did not involve wild animals.
Reporting on sex	To ensure gender uniformity and reduce aggression-related complications, only female mice were used in this study, as male mice are more prone to fighting when housed together. However, since the analysis focuses on comparisons between treated and untreated groups, the sex of the host is considered to have minimal impact on the outcomes. Therefore, the experiment was designed without accounting for sex as a biological variable.
Field-collected samples	No Field-collected sample were used in this study
Ethics oversight	All mice studies were approved (protocol #19-545) by UArizona IACUC and followed ethical guidelines. Mice were euthanized per IACUC guidelines if tumours reached maximal permitted size 2000 mm3 or if moribund (severe weight loss, weakness, or inactivity). In rare cases, tumours slightly exceeded 2000 mm3 on the final measurement day but were promptly addressed.

Note that full information on the approval of the study protocol must also be provided in the manuscript.

Plants

Seed stocks	NA
Novel plant genotypes	NA
Authentication	NA

Flow Cytometry

Plots

Confirm that:

- [x] The axis labels state the marker and fluorochrome used (e.g. CD4-FITC).
- [x] The axis scales are clearly visible. Include numbers along axes only for bottom left plot of group (a 'group' is an analysis of identical markers).
- [x] All plots are contour plots with outliers or pseudocolor plots.
- [x] A numerical value for number of cells or percentage (with statistics) is provided.

Methodology

Sample preparation	Cells were washed, trypsinized, isolated by centrifugation, and resuspended in 500 µL of staining buffer.
Instrument	BD FACSCanto II (BD FACSDiva Software, version 8.01)
Software	BD FACSDiva Software (version 8.01) was used for data collection, FlowJo software (version 10.0.7, TreeStar, USA, 2014) was used for data analysis.
Cell population abundance	No sorting was performed.
Gating strategy	Generally, cells was first gated on FSC/SSC. Singlet cells were usually gated using FSC-H and FSC-A and live cells were gated using Zombie violet .

- [x] Tick this box to confirm that a figure exemplifying the gating strategy is provided in the Supplementary Information.
